# Harnessing Selectivity in CO_2_ Hydrogenation: Mechanistic Descriptors, Data-Driven Catalyst Design, and Sustainability Constraints

**DOI:** 10.1021/acsenvironau.6c00048

**Published:** 2026-07-01

**Authors:** Rahul Mishra, Yu-Chuan Lin

**Affiliations:** † Department of Chemical Engineering, 34912 National Cheng Kung University, Tainan 70101, Taiwan; ‡ Center for Resilience and Intelligence on Sustainable Energy Research (RiSER), National Cheng Kung University, Tainan 70101, Taiwan; § Program on Smart and Sustainable Manufacturing, Academy of Innovative Semiconductor and Sustainable Manufacturing, National Cheng Kung University, Tainan 70101, Taiwan

**Keywords:** artificial intelligence and machine learning, CO_2_ hydrogenation, life cycle assessment, methanation, operando characterization, reverse water−gas shift, selectivity control, techno-economic analysis

## Abstract

Rising CO_2_ emissions continue to motivate catalytic strategies that recycle carbon into useful products. In CO_2_ hydrogenation, environmental relevance depends not only on conversion but on selectivity control and operational stability. The reverse water–gas shift, methanation, methanol synthesis, and C_2_^+^ pathways operate within overlapping temperature and pressure windows, where minor changes in catalyst state or reaction conditions can markedly alter product distribution and downstream separation requirements. This review frames CO_2_ hydrogenation as a selectivity-driven design challenge. Key reaction intermediates governing pathway competition, including adsorbed CO, formate, methoxy species, and carbide phases, are identified, and recurring selectivity levers across catalyst families are discussed. Operando and in situ studies are highlighted for resolving working states and dynamic restructuring under reaction conditions. Beyond mechanistic insights, emerging artificial intelligence and machine learning approaches are examined for accelerating catalyst and operating window identification. Techno-economic analysis and life cycle assessment perspectives are integrated to link selectivity to separation energy, process efficiency, and net climate impact. This framework supports environmentally informed evaluation and deployment of CO_2_ hydrogenation technologies.

## Graphical abstract

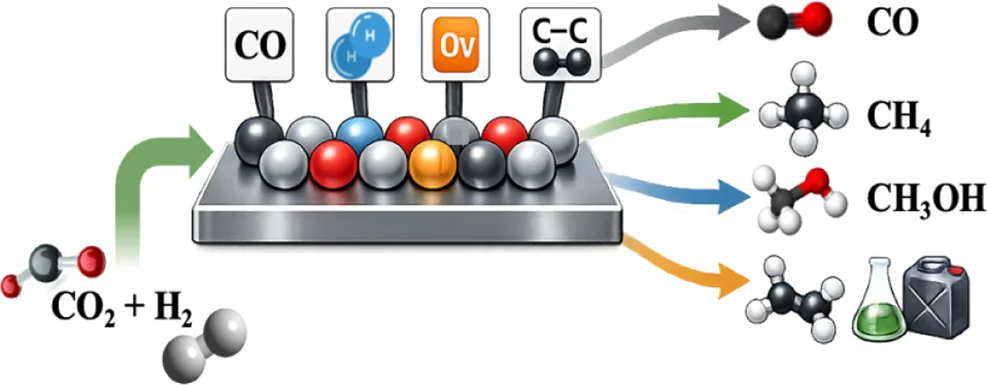

## 1 Introduction

CO_2_ hydrogenation is increasingly discussed as a strategy for carbon utilization because it combines captured CO_2_ with renewable H_2_ to produce fuels and essential chemicals, using industrially scalable processes.^1,2^ However, this field is evaluated not by the feasibility of CO_2_ conversion but by the ability to achieve selective and stable operation with meaningful climate benefit under practical constraints.^1^ Selectivity dominates because CO_2_ hydrogenation is a coupled reaction network in which reverse water gas shift (RWGS), methanation, methanol synthesis, and hydrocarbon synthesis can compete within the same operating window.^3,4^ Even for C_1_ products, small changes in catalyst composition, support chemistry, and defect structure, or hydrogen availability can redirect the same CO_2_/H_2_ feed toward CO, CH_4_, or oxygenates, thereby dictating separation demand, recycle requirements, and overall process efficiency.^3^ The selectivity control is therefore a design challenge, where catalysts are engineered to regulate a limited set of rate-determining and branch-controlling steps rather than optimized through empirical screening.^3^

Many selectivity trends can be traced back to a few core mechanistic knobs. CO_2_ can be activated through redox and/or associative routes,^3–5^ and product branching then depends on how strongly CO-derived intermediates bind, how effectively H_2_ supplies surface H, and how oxygen vacancies (Ov) or defects assist oxygen removal and stabilize key intermediates.^3,5^ These knobs are coupled, so improving one function, e.g., hydrogenation ability, can unintentionally promote or suppress competing pathways, e.g., shifting the balance between RWGS and methanation, making robust selectivity difficult to maintain.^3,4^

From a materials perspective, most practical catalysts are solid-supported and structurally heterogeneous, so selectivity depends on active-phase dispersion, interface design, promoter effects, and how the catalyst evolves under water-rich and thermally demanding conditions.^2,6^ Operando and in situ studies show that the working catalyst can differ from the structure identified ex situ, and restructuring can change the active sites and shift selectivity over time, e.g., Fe-based systems in C_2_^+^ production.^6^ Selectivity becomes more challenging for multicarbon products because C–C coupling must be promoted without falling into broad product distributions of chain-growth chemistry.^5,7^ In this context, tandem catalysis represents a conceptual advance, referring to catalytic systems in which multiple active sites or catalyst components sequentially facilitate different reaction steps. It enables selective design by selecting intermediates and arranging complementary catalytic functions, so that the overall system follows a controlled pathway rather than a stochastic outcome.^5^ This modular logic explains why high-performing CO_2_-to-C_2_^+^ systems behave as integrated catalyst ensembles, where activation, intermediate formation, and C–C chemistry are assigned to distinct functions.^5,7^

Finally, selectivity must be interpreted in the context of systems and sustainability. The real carbon benefit and competitiveness of CO_2_ hydrogenation depend strongly on CO_2_ sourcing, hydrogen carbon intensity, heat and water integration, and downstream separation penalties.^1^ Unlike previous reviews that treat mechanisms, catalysts, or processes separately, this work presents CO_2_ hydrogenation as a selectivity-driven design challenge, emphasizing how catalyst properties and reaction conditions govern competing pathways and product distribution. This review frames CO_2_ hydrogenation as a selectivity-design challenge across three interconnected layers: mechanism, data, and deployment. First, it unifies the field around key mechanistic factors that govern reaction pathway branching, including CO_2_ activation, CO adsorption and conversion, hydrogen coverage, and defect or interface effects, thereby explaining selectivity outcomes. Second, it treats artificial intelligence (AI) and machine learning (ML) as a design tool, highlighting how descriptors, surrogate models, and closed-loop workflows can accelerate the discovery of catalyst compositions and operating windows. Third, it places catalytic claims within a techno-economic analysis (TEA) and life cycle assessment (LCA) reality check by linking selectivity targets to separation work, recycling intensity, heat management, electricity use, H_2_-carbon intensity, and CO_2_ sourcing assumptions. Thus, the best catalysts are judged by deployability and net impact, not only by lab-scale conversion and selectivity. This framework connects mechanistic insight with data-driven optimization and system-level constraints to guide catalyst–reactor–separation codesign for practical CO_2_ hydrogenation.

## 2 Reaction Network and Selectivity

### 2.1 Overview of Parallel Routes of RWGS, Methanation, and Syngas to Hydrocarbons

RWGS catalysis is a critical process for producing CO from CO_2_ and H_2_ (Reaction 1), providing a bridge between carbon capture and utilization (CCU), green hydrogen production, and synthesis-gas-based chemical processes such as methanol, acetic acid, and Fischer–Tropsch hydrocarbons.^8,9^

1 $$CO_{2}+H_{2}⇋CO+H_{2}O\;\Delta H=+41\;kJ/mol$$

The RWGS mechanism depends strongly on catalyst type and operating conditions, with two principal pathways proposed: the redox route and the associative route, the latter subdivided into formate and carboxylate pathways (Figure 1). In the redox mechanism (orange in Figure 1), CO_2_ adsorbs and dissociates at electron-donating sites, such as metal centers or Ov in reducible oxides. The resulting *CO desorbs, while the remaining oxygen is hydrogenated to H_2_O via surface *H, regenerating the active site.^10^ CO_2_ can also reoxidize metal sites, thereby sustaining the redox cycle and promoting continuous CO production. The associative mechanism proceeds via hydrogen-assisted intermediates. In the formate pathway (green), CO_2_ reacts with adsorbed hydrogen (*H) to form surface *HCOO, which subsequently decomposes to CO.^10^ In the carboxyl pathway (blue), CO_2_ and *H generate *COOH intermediates (cis- or trans-isomers), which break down into CO and H_2_O.^11–13^ The key distinction between the redox and associative routes is whether H_2_ dissociation actively contributes to the formation of carbon-containing intermediates. Both mechanisms can coexist, with relative dominance determined by catalyst composition and reaction conditions.^14,15^ The elementary steps are as follows:

2 $$H_{2}+2^{*}\to 2^{*}H$$

3 CO2+*→CO2*

4 CO2*+*→CO*+O*

5 H*+O*→OH*+*

6 OH*+OH*→H2O*+O*

CO2*+H*→HCOO*+* 7

HCOO*+*→HCO*+O* 8

HCO*+*→CO*+OH* 9

HCOO*+*→CO*+OH* 10

11 CO2*+H*→COOH*+*

12 COOH*+*→CHO*+O*

13 COOH*+*→CO*+OH*

14 H*+OH*→H2O*

15 H2O*→H2O+*

16 CO*→CO+*

**Figure 1:**
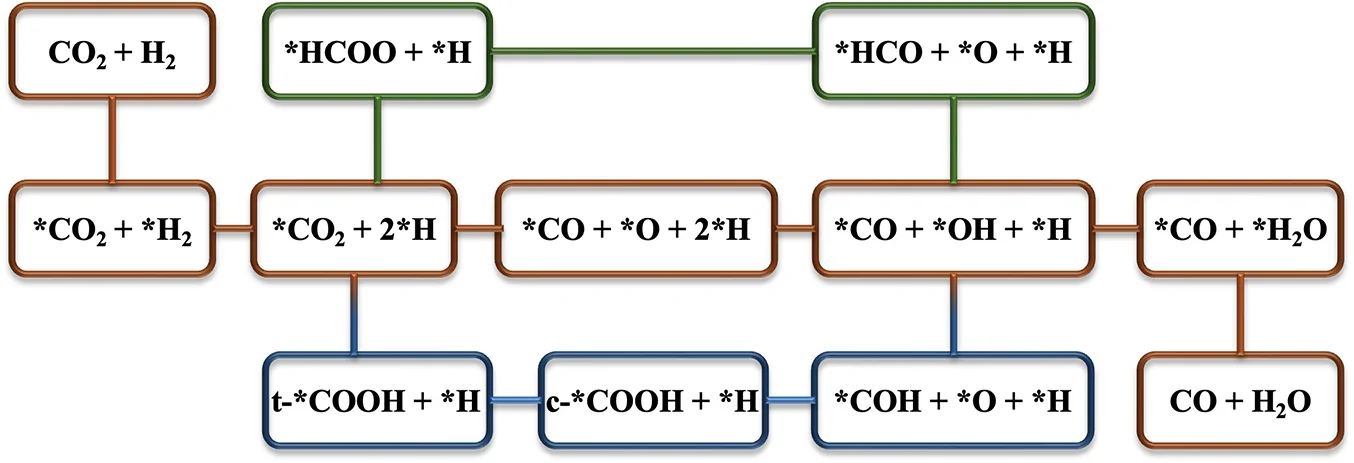
Schematic representation of RWGS reaction pathways: the redox route (orange), the associative formate route via HCOO intermediates (green), and the associative carboxyl route via COOH intermediates (blue, including cis- and trans-carboxyl species).

The catalytic hydrogenation of CO_2_ to CH_4_ is the primary step in CO_2_ methanation, also known as the Sabatier reaction. This exothermic reaction (Reaction 17) is accompanied by several secondary routes, including the RWGS (Reaction 1) and CO hydrogenation (Reactions 18 and 19):^16^

17 $$CO_{2}+4H_{2}⇋CH_{4}+2H_{2}O\;\Delta H=-165\;kJ/mol$$

18 $$CO+3H_{2}⇋CH_{4}+H_{2}O\;\Delta H=-206\;kJ/mol$$

19 $$2CO+2H_{2}⇋CH_{4}+CO_{2}\;\Delta H=-247\;kJ/mol$$

CO_2_ methanation is exothermic and thus favored thermodynamically at low temperatures. However, reaction temperatures above 200 °C are required to overcome the high activation barrier of C=O bond dissociation. At higher temperatures (>450 °C), side reactions such as RWGS become dominant, reducing CO_2_ conversion efficiency. Experimental studies show that conversion decreases with increasing temperature, reaching a minimum at 550–600 °C before rising again. This behavior reflects the growing influence of RWGS, which defines the practical operating window for CO_2_ methanation as 200–450 °C.^17^ Figure 2a highlights three main routes toward methane formation: the C–O cleavage pathway, the RWGS–CO route, and the formate-mediated route. Each involves distinct surface intermediates such as *CO, *CHO, *COOH, *CH_*x*_, and CH_*x*_OH, which undergo successive hydrogenation steps to yield CH_4_. In contrast to methanation, methanol synthesis proceeds predominantly through associative pathways involving formate (*HCOO) and methoxy (*CH_3_O) intermediates.^18^ CO_2_ is first hydrogenated to form surface formate species, which are subsequently reduced through formaldehyde-like intermediates to methanol. The stabilization of oxygenated intermediates and controlled hydrogenation activity are critical for directing selectivity toward methanol rather than complete hydrogenation to methane (Figure 2a). These pathways highlight a key selectivity divergence: methanation generally involves deep hydrogenation of CO-derived intermediates, whereas methanol synthesis proceeds via the stabilization of partially hydrogenated oxygenates. Accordingly, catalysts exhibiting relatively strong CO binding and high hydrogenation activity (e.g., Ni-based systems) tend to favor methane formation, while catalysts that stabilize formate and methoxy intermediates (e.g., Cu-based systems) are widely reported to promote methanol synthesis.^4,10,19^

**Figure 2:**
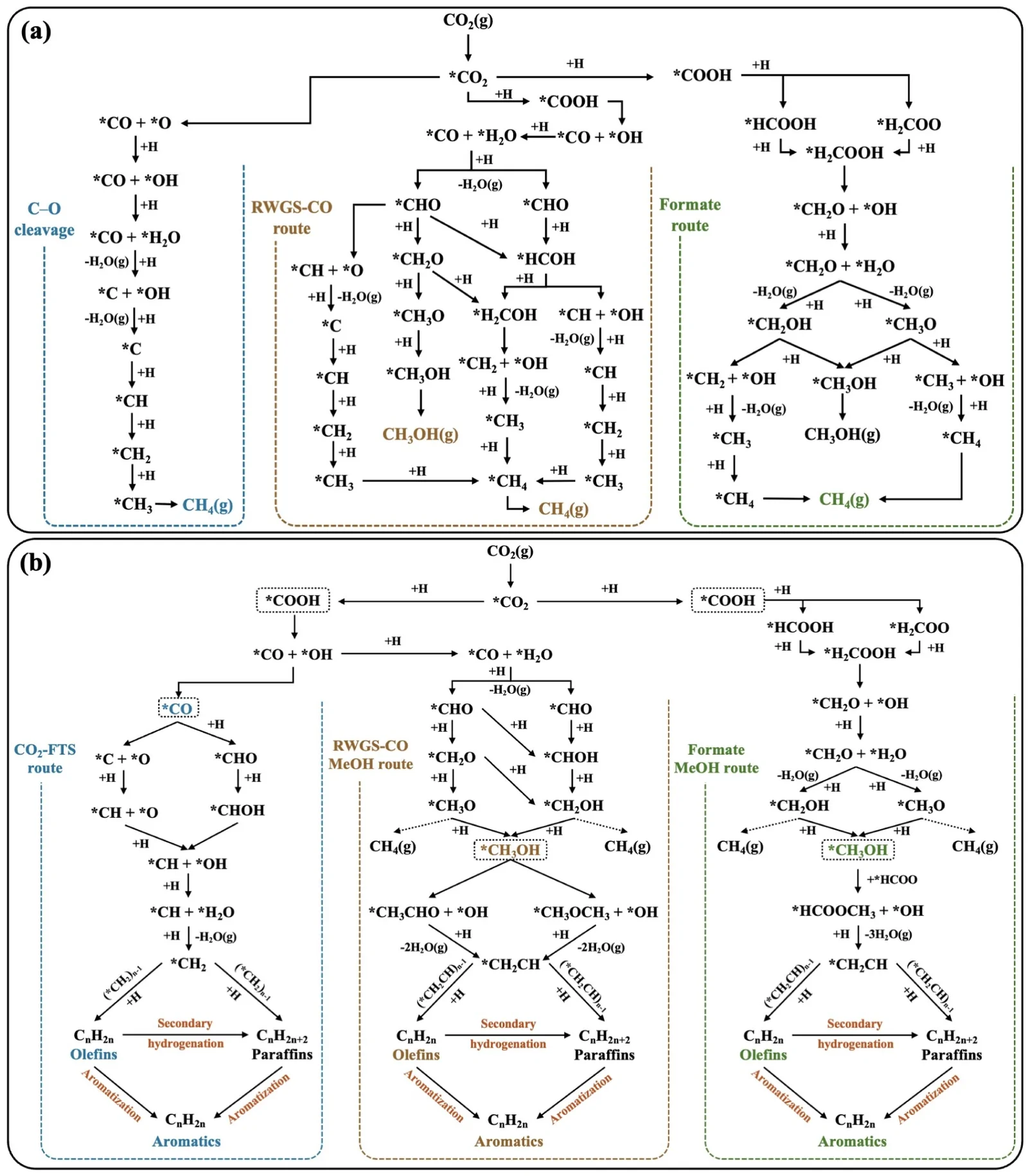
Reaction pathway of CO_2_ hydrogenation to (a) CH_4_ and CH_3_OH. Adapted with permission from ref 18. Copyright 2024, Wiley. (b) Olefins, aromatics, and other hydrocarbons. Adapted with permission from ref 27. Copyright 2021, Elsevier.

Parallel and sequential pathways enable the CO_2_–FTS reaction to produce a range of hydrocarbons. The generation of C_2_^+^ hydrocarbons is generally attributed to two sequential phases, although some studies propose an alternative, albeit minor, direct pathway for greater hydrocarbon formation from CO_2_ without CO acting as an intermediate.^20,21^ In the first step, CO_2_ is hydrogenated to CO using the RWGS reaction (Reaction 1). FTS forms C–C bonds (Reactions 20 and 21), while secondary hydrogenation of olefins is the primary process for paraffin synthesis (Reaction 22).^22,23^

20 $$nCO+2nH_{2}⇋C_{n}H_{2n}+nH_{2}O$$

21 $$nCO+\left(2n+1\right)H_{2}⇋C_{n}H_{2n+2}+nH_{2}O$$

22 $$C_{n}H_{2n}+H_{2}⇋C_{n}H_{2n+2}$$

Byproduct CH_4_ is produced either from CO_2_ methanation or from CO methanation and hydrogenolysis of C_2_^+^ hydrocarbons, based on the catalyst composition and reaction conditions.^24^ The degree of CO_2_ conversion significantly affects product selectivity due to the intricate reaction network of CO_2_–FTS. Several reactions involved in CO_2_–FTS or MeOH-mediated processes are shared with CO_2_ hydrogenation to olefins and aromatics.^25^ Figure 2b illustrates that CO_2_–FTS and methanol-mediated (MeOH) routes are the main pathways to olefins and paraffins, which can undergo cracking, cyclization, and dehydrogenation to form aromatics. In this scheme, CO generated via RWGS is essential for the CO_2_–FTS pathway. Thermodynamically, CO_2_–FTS is favored over RWGS alone, as FTS is exothermic while RWGS is endothermic. Moreover, the consumption of CO in FTS drives the RWGS equilibrium toward further CO production when both reactions occur concurrently.^26^

### 2.2 Key Intermediates (Chemisorbed CO, Formate, Methoxy, Carbide)

#### 2.2.1 Chemisorbed CO

As the branching point between the formation of syngas through RWGS and further hydrogenation to CH_4_ or higher hydrocarbons, chemisorbed CO (*CO) is an essential intermediate in CO_2_ hydrogenation. However, its mechanistic role remains the subject of debate. For example, in Ni catalysts, constant *CO coverage was observed along the catalyst bed by operando DRIFTS on Ni/γ-Al_2_O_3_, indicating a spectator role.^28^ In contrast, tightly bound carbonyls (linear, bridged, and multibonded *CO) undergo direct hydrogenation to CH_4_, shown by isotopic transient DRIFTS on Ni/SiO_2_, indicating *CO is a reactive intermediate.^29^ *CO’s reactivity is structure sensitive. At the same time, CO methanation necessitates step-edge sites; RWGS-derived CO synthesis is relatively insensitive to Ni particle size, which explains why nanoparticles smaller than 5 nm inhibit CH_4_ formation and promote RWGS.^29^ In addition to Ni, the Cu-based system emphasizes another aspect of CO’s role. In situ X-ray spectroscopy revealed that CO scavenges adsorbed oxygen species, thereby regenerating metallic Cu sites essential for CO_2_ activation and sustaining methanol synthesis, highlighting its dual role as an intermediate and an oxygen regulator.^30^ Other studies also highlight that the balance between the desorption and hydrogenation rates of *CO, which depends on the electronic structure of the catalyst and the site environment.^31,32^ Noble metals such as Pt, Pd, and Au tend to favor CO desorption, while Ni, Co, and Ru promote deeper hydrogenation to CH_4_.^33^ Specific metal–support interactions, like Ni clusters on hydroxylated TiO_2_, induce high CO_2_ conversion and CO selectivity by promoting the CO desorption rate. The high CO selectivity of single-atom and subnanometer catalysts is attributed to the decreased bonding strength of CO.^34^ The regulation of *CO is essential to catalyst design because it is a dual-role species whose behavior, desorption as CO or hydrogenation to CH_4_, is determined by particle size, site availability, electronic structure, and support interactions.

#### 2.2.2 Formate

In CO_2_ hydrogenation, particularly in methanol synthesis, the formate (*HCOO) species is frequently identified. Most studies claim the pathway in which CO_2_ reacts with surface atomic H to form *HCOO, followed by sequential hydrogenation to dioxomethylene (*H_2_COO), formaldehyde (*CH_2_O), methoxy (*CH_3_O), and finally CH_3_OH.^35^ Still, the mechanistic relevance of *HCOO is debated. Due to the substantial activation barriers associated with formate’s hydrogenation, some research suggests that formate may function as a bystander, despite being frequently observed in operando spectroscopies.^36^ Indium oxide (In_2_O_3_)-based catalysts are especially associated with the formate route, as oxygen vacancies facilitate CO_2_ activation and stabilize *HCOO intermediates, thereby enhancing methanol selectivity.^37^ According to Sun et al.,^38^ heterogeneous catalysts across different scales, from single atoms to nanoparticles, stabilize formate species in distinct ways, with atomically dispersed sites offering unique opportunities to tune their reactivity and conversion. More comprehensive mechanistic evaluations find that the formate pathway is frequently preferred on reducible oxides and metal–oxide interfaces, despite competing with the carboxyl (*COOH) pathway.^1,32^ These results indicate that the formate pathway is crucial in driving CO_2_ conversion, regardless of the catalyst’s structure and reaction environment.

#### 2.2.3 Methoxy

A crucial step in the hydrogenation of CO_2_, particularly in the methanol-mediated pathway, is the formation of the methoxy (*CH_3_O) species. CO_2_ is reduced subsequently to formate (*HCOO), formaldehyde (*CH_2_O), and dioxymethylene (*H_2_COO), and then to methoxy (*CH_3_O). Formic acid (*HCOOH) is produced by preferentially hydrogenating formate species, which are subsequently converted to *H_2_COOH and then to produce formaldehyde and OH species. Eventually, a methoxy species was coupled with a formaldehyde species to form methanol.^39^ Hong et al.^40^ report that formate and carbonyl species are produced through the interaction of CO_2_ with active hydrogen species, which may begin a variety of hydrogenation pathways. Therefore, methanol production via CO_2_ hydrogenation depends on the dominant H_2_ activation pathway and sufficient surface hydrogen coverage (*H) to enable selective hydrogenation. Methoxy species are essential for the conversion of CO_2_-derived methanol to light hydrocarbons in bifunctional oxide–zeolite systems, in addition to methanol production. Surface methoxy species, which are thought to be crucial intermediates in methanol-to-hydrocarbons, are produced when methanol is dehydrated in zeolites.^41^ The hydrocarbon-pool mechanism underpinning both methanol-to-olefins and methanol-to-aromatics relies on surface methoxy groups, which readily transfer methyl groups to aromatics and olefins. Zhang and Wei^42^ reported that the methanol-intermediate route can overcome the limitation imposed by the Anderson–Schulz–Flory rule and enhance the selectivity of light olefins in hydrocarbons to 70%–87%. Methoxy species on Brønsted acid sites, which channel C_1_ units into C–C linked products, are responsible for this enhancement. These observations make methoxy a crucial component of CO_2_ hydrogenation, enabling the conversion of methanol to light olefins and aromatics and facilitating the conversion of formate and formaldehyde to methanol. The nature of the catalyst can direct the methoxyl transformation in different ways. Zeolitic Brønsted acid sites convert *CH_3_O into higher hydrocarbons, Cu–ZnO interfaces promote the synthesis of methanol, and In_2_O_3_ surfaces stabilize *COOH and *CH_3_O.

Beyond isolated discussions of individual intermediates, recent studies emphasize that their relative stability and transformation are strongly governed by site-dependent energetics across the catalyst surface. Recent density functional theory (DFT) studies have provided important insights into the structure-dependent activity of catalysts during CO_2_ hydrogenation. For example, Hu et al.^43^ demonstrated that on MoS_2_, CO_2_ preferentially adsorbs and dissociates at sulfur vacancy (Sv) sites, particularly in the in-plane configuration, forming *CO and *O with low activation barriers (<0.33 eV). In contrast, associative pathways via *COOH or *HCOO intermediates are less favorable at these sites, in contrast to conventional metal and metal–oxide catalysts. Following CO_2_ dissociation, *CO is more likely to undergo hydrogenation to *CHO rather than desorption, and subsequent hydrogenation through *CH_2_O and *CH_3_O intermediates proceeds with comparatively lower barriers, favoring methanol formation (Figure 3). Importantly, the catalytic behavior is strongly site dependent. In-plane Sv sites facilitate methanol production due to favorable CH_3_OH desorption, whereas Mo-edge Sv sites promote methanol dissociation to *CH_3_ and *OH, making methane formation more competitive. These findings are consistent with operando spectroscopic observations, which show the evolution of *CH_3_O species from *CO intermediates. Overall, this study highlights that selectivity in CO_2_ hydrogenation is governed not only by reaction energetics but also by the nature of active sites and their associated adsorption–desorption characteristics.

**Figure 3:**
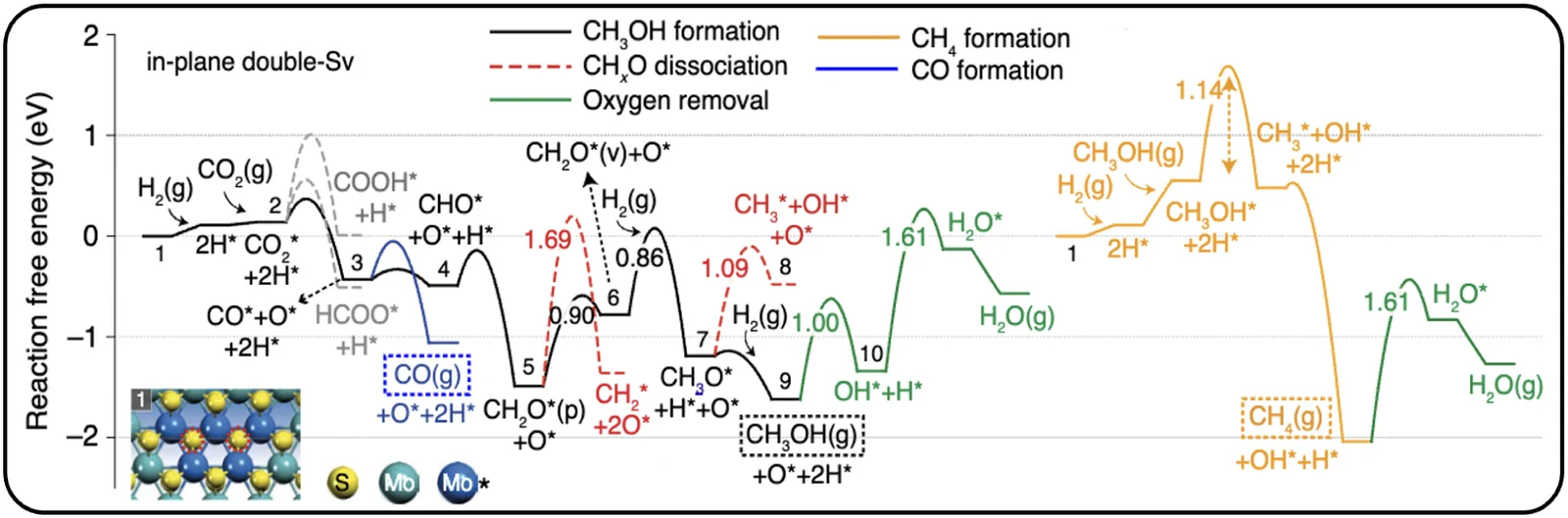
Representative free energy profiles for CO_2_ hydrogenation on MoS_2_ illustrating competing reaction pathways toward CO, CH_4_, and CH_3_OH. The figure highlights the roles of key intermediates (*CO, *CHO, *CH_2_O, and *CH_3_O), the rate-determining steps, and the influence of sulfur vacancy (Sv) sites on pathway selectivity. Adapted with permission from ref 43. Copyright 2021, Springer Nature.

#### 2.2.4 Carbide

In CO_2_ hydrogenation, carbide species are active in Fischer–Tropsch-like processes. For example, in CO_2_–FTS, iron carbide (Fe_5_C_2_) is proposed to be the active phase.^25^ Metal carbides, such as χ-Fe_5_C_2_, can be formed when adsorbed CO dissociates and diffuses into the iron oxide lattices.^44^ In general, the electronic structures of transition metal carbides (TMCs) resemble those of noble metals. TMCs exhibit electronic configurations and catalytic behavior similar to those of noble metals.^18^ The ability of χ-Fe_5_C_2_ to activate CO_2_ is linked to its carbide nature, as incorporation of carbon into the Fe lattice alters the electronic structure and facilitates CO_2_ adsorption and C–O bond cleavage. CO_2_ was nondissociatively activated on χ-Fe_5_C_2_ to produce CH_4_, indicating that the carbide phase can actively participate in CO_2_ hydrogenation rather than serving as a carbon sink. Molybdenum carbides have also been identified as effective catalysts for CO_2_ hydrogenation, with activity comparable to that of noble metals.^45^ Ye et al.^1^ emphasized that the primary factor determining the catalysts’ catalytic activity is the nature of the active site. Metallic sites are efficient for H_2_ dissociation and tend to yield CO or CH_4_. Oxide sites, on the other hand, stabilize oxygenated intermediates such as formate and methoxy, which promote methanol formation. Meanwhile, carbide phases can facilitate C–O bond cleavage and stabilize C species, enabling CH_4_ formation and Fischer–Tropsch–like chain growth. These studies demonstrate that carbide phases can store and transmit *C species, thereby controlling whether CO_2_ conversion yields CO, CH_4_, or longer hydrocarbons.

### 2.3 Selectivity Levers

#### 2.3.1 CO Binding Strength

Among the various descriptors proposed for CO_2_ hydrogenation, CO binding strength (i.e., the adsorption strength of chemisorbed CO on the catalyst surface) has emerged as a central parameter because it dictates whether CO acts as a final product or a reactive intermediate.^1,3,25^ Alongside the C–O bond dissociation energy, CO binding governs the reaction pathway and ultimately dictates selectivity toward CO, methanol, or CH_4_ (Figure 4).^3,9^ Weak CO binding favors CO desorption via the RWGS pathway; moderate binding enables hydrogenation to methanol; and strong binding stabilizes CO, promoting methane formation.^3,9,25^ The ability to break C–O bonds further modulates these pathways, with moderate C–O dissociation supporting methanol formation, whereas strong dissociation favors deeper hydrogenation to CH_4_.^1,9,10^ The role of *CO has been extensively investigated on different catalysts, each showing distinct adsorption characteristics. At Cu–ZnO interfaces, CO binds moderately, allowing for either desorption or further hydrogenation to methanol, which explains the high methanol selectivity observed.^19,37^ Ni catalysts, particularly at step-edge sites, bind CO strongly, thereby stabilizing it and promoting CH_4_ formation.^3^ Particle size effects are also critical, as smaller Ni nanoparticles suppress methanation due to weaker CO binding.^29^ Fe-based catalysts behave differently because CO adsorption leads to either syngas formation or dissociation into carbides, which enable C–C coupling and hydrocarbon growth.^44^ These insights highlight CO binding strength and C–O bond dissociation as unifying selectivity levers across catalyst families. Selectivity toward CO, methanol, or methane can be optimized by tuning particle size, site type, support interactions, or alloying.

**Figure 4:**
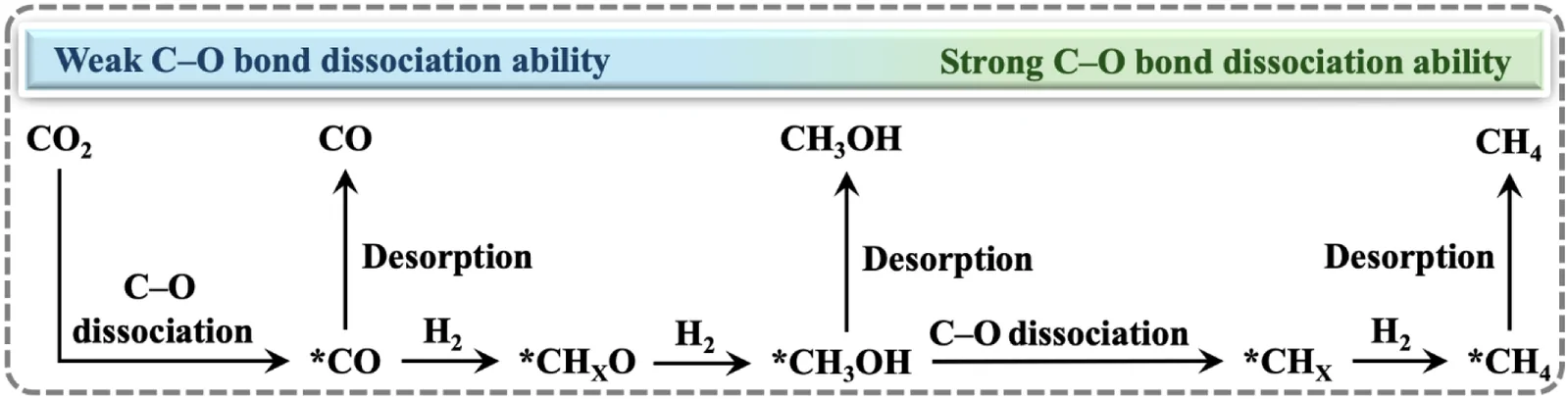
Consecutive reaction pathway illustrating the influence of C–O dissociation, hydrogenation, and CO desorption on the selectivity of CO_2_ hydrogenation. Adapted with permission from ref 3. Copyright 2024, Elsevier.

#### 2.3.2 H* Coverage and H_2_ Activation Processes

In CO_2_ hydrogenation, a catalyst’s ability to dissociate H_2_ and generate reactive hydrogen species (*H) is a key selectivity lever, as it controls surface hydrogen coverage and determines the competition between partial and complete hydrogenation pathways. Weak H_2_ activation limits *H availability, favoring the stabilization of oxygenated intermediates such as formate and methoxy, which explains the high methanol selectivity observed on Cu-based catalysts, particularly at Cu–ZnO and Cu–In_2_O_3_ interfaces.^19,37,46^ In contrast, metals such as Ni, Ru, and Co exhibit strong H_2_ dissociation, leading to high *H coverages that accelerate complete hydrogenation and promote CO_2_ methanation.^29,47^ Structure sensitivity further modulates this behavior; for example, on Ni, terrace sites provide moderate hydrogenation activity, while step-edge sites with higher *H densities favor rapid CH_4_ formation.^48^ In oxide-based systems like In_2_O_3_ and CeO_2_, methanol selectivity arises from the interplay between *H supply and CO_2_ activation at oxygen vacancies, where H_2_ activation occurs either via spillover from metal sites or through vacancy-assisted pathways.^1,37^ This establishes a unifying principle: surface hydrogen coverage (*H), as a measure of hydrogen activation, governs product distribution, with high *H coverage favoring CH_4_ formation and lower coverage promoting oxygenates such as methanol.^3,17,49^ This selectivity lever can be tuned in practice by introducing promoters that block overactive hydrogenation sites, selecting supports that modulate H_2_ spillover (e.g., Ni/CeO_2_), or combining Cu with more active hydrogenation metals (e.g., Cu–Ni, Cu–Zn), thereby controlling the balance between *H supply and intermediate stabilization.^3,50^

#### 2.3.3 Oxygen Vacancies

Oxygen vacancies (Ov) are defects in reducible oxides, which alter both bulk properties, such as conductivity and electronic structure, and surface properties, like adsorption sites and composition.^51^ Engineering Ov has emerged as a key to enhancing catalytic performance in thermocatalysis. In CO_2_ hydrogenation, Ov acts as a site for CO_2_ adsorption and the transformation of surface intermediates.^52–54^

Recent studies show a positive correlation between Ov density and catalytic activity. Pd catalysts supported on CeO_2_ rods, which possess higher Ov density than other morphologies, exhibited enhanced CO_2_ adsorption and methanol formation.^55^ Similarly, Cu/ZnO/ZrO_2_ catalysts with abundant Ov increased methanol productivity.^56^ A linear relationship between Ov density and CH_4_ production over inverse CeAlO_*x*_/Ni catalysts was also observed, highlighting the role of interfacial Ov in CO_2_ methanation.^57^ Unpaired electrons at Ov on catalysts such as ZnGa_2_O_*x*_ and ZnAl_2_O_*x*_ facilitated CO_2_ dissociation and hydrogenation to methanol,^58^ while La-doping in NiCeO_*x*_ increased Ov content, promoting H_2_ dissociation for hydride formation.^59^ Zhang et al.^60^ showed that Ov-rich ZnO_1–x_/Cu reduced the energy barrier for *COOH → H_2_COO conversion, enhancing methanol formation. Single-atom Co or La-doped Ni/CeO_2–x_ catalysts with abundant Ov improved CO_2_ adsorption, stabilized COOH intermediates, and facilitated low-temperature methanation.^61,62^ Furthermore, interfacial Ov at Ni/In_2_O_3_ and Ru/MnO enhanced hydrogenation pathways, directly influencing selectivity toward methanol or CH_4_.^63,64^ Previous studies establish that Ov is pivotal in CO_2_ adsorption, activation, and intermediate stabilization. Tailoring the oxygen density and distribution is important for tuning activity and selectivity for methanol, CO, or CH_4_ production.

#### 2.3.4 C–C Coupling Pathways

Unlike other descriptors, C–C coupling is treated here as a mechanistic step linked to kinetic parameters governing chain growth. C–C coupling is a key step in the formation of multicarbon products, such as ethylene and ethanol. The reaction involves the combination of C_1_ intermediates (*CO, *CHO) on catalyst surfaces, with the pathway and energetics of coupling strongly influencing product selectivity.^65^ On Cu-based catalysts, *CO dimerization leads to *CO*CO intermediates, which are protonated and reduced to *CO*COH. These species can evolve into *CH*COH or CH_2_CH*O. The hydrogenation of CH_2_CH*O produces C_2_H_4_, while further hydrogenation to CH_3_CH*O yields C_2_H_5_OH. Energy calculations indicate that ethylene formation via CH_2_CH*O is energetically favored over ethanol formation, which explains the higher ethylene selectivity observed on many Cu catalysts.^66,67^

Alternative pathways include direct coupling of *CO and *CHO to form *COCHO intermediates, which, upon further hydrogenation, produce *OCHCH*O and CH_2_CH*O, ultimately yielding ethylene.^65^ Dimerization of *CHO intermediates via proton-coupled electron transfer (PCET) provides a third pathway, with lower energy barriers than *CO–*CHO coupling, highlighting the preferential formation of C–C bonds through *CHO intermediates.^68,69^

Recent studies have demonstrated that catalyst structure and surface engineering strongly influence C–C coupling efficiency. High-index facets on Cu_2_O nanoparticles, such as (332) and (111), reduce the Gibbs free energy barriers for intermediates, thereby enhancing the selectivity for ethylene or ethanol.^70,71^ Defect engineering, such as CuOCu–N_4_ binuclear sites or nanoscale defects in Cu nanosheets, stabilizes intermediates and lowers the C–C coupling barriers, thereby increasing the faradaic efficiency for C_2_ products.^72,73^ Oxidation state control stabilizing Cu^+^ in ultrathin Cu_2_O nanosheets, optimizes *CO adsorption, increases active site density, and promotes selective C–C coupling.^74^ These studies demonstrate that precise control over surface structure, defects, and oxidation state is crucial for enabling efficient C–C coupling, thereby providing a robust strategy for selectively producing multicarbon products in CO_2_ reduction.

### 2.4 Section Summary and Design Implications

Across RWGS, methanation, methanol synthesis, and C_2_^+^ pathways, selectivity in CO_2_ hydrogenation is governed by a recurring set of mechanistic descriptors rather than isolated reaction routes. In particular, CO binding strength, surface hydrogen coverage resulting from H_2_ activation, oxygen-vacancy chemistry, and C–C coupling kinetics collectively determine pathway competition and product distribution. These descriptors are strongly interdependent, such that tuning one often shifts the balance between parallel reactions. Therefore, rational catalyst design requires controlling these coupled parameters under realistic reaction environments, where dynamic restructuring, water formation, and thermal effects can modify active sites. These mechanistic insights establish a unified descriptor-based framework that can be directly translated into data-driven catalyst design strategies.

Because product selectivity in CO_2_ hydrogenation is strongly dependent on operating conditions and conversion level, selectivity values should not be compared in isolation. Temperature, pressure, H_2_/CO_2_ ratio, space velocity, residence time, and reactant conversion can all shift the relative contributions of RWGS, methanation, methanol synthesis, and C_2_^+^ formation. Therefore, throughout this review, selectivity data are discussed together with the corresponding conversion and reaction conditions whenever available. Comparisons across catalyst families should be interpreted cautiously unless the reported data were obtained under comparable conversion regimes and operating windows.

## 3 Catalyst Paradigms for Selectivity Control

### 3.1 Ni-Based Systems for CO vs CH_4_ Selectivity

Nickel-based catalysts remain the cornerstone of CO_2_ hydrogenation owing to their activities in C–O bond and H_2_ dissociation.^75^ However, the selectivity between CO and CH_4_ is sensitive to catalyst structure, support characteristics, and reaction temperature. The competition between CO desorption and subsequent hydrogenation dictates whether CO or CH_4_ is the dominant product.

Mechanistically, two parallel routes proceed on Ni surfaces: the *CO pathway and the *COOH pathway. In the *CO pathway, CO_2_ first dissociates to *CO, which is then sequentially hydrogenated to CH_4_. In contrast, in the *COOH pathway, CO_2_ reacts with surface *H species to form *COOH, which is then reduced to CH_4_ (Figure 5a).^75^ In situ DRIFTS and DFT analyses revealed that Ni(111) facets are the most active for H_2_ dissociation and CO_2_ hydrogenation, as shown in Figure 5b.^76^ The reaction begins with hydrogenation at the carbon terminus of adsorbed *CO_2_, forming *COOH, followed by O-terminal hydrogenation to *HCOOH. Cleavage of the C–O bond and removal of surface *OH yield *HCO, which undergoes stepwise hydrogenation to *CH_2_, *CH_3_, and ultimately *CH_4_, accompanied by surface H_2_O formation through *OH + *H coupling.^76^

**Figure 5:**
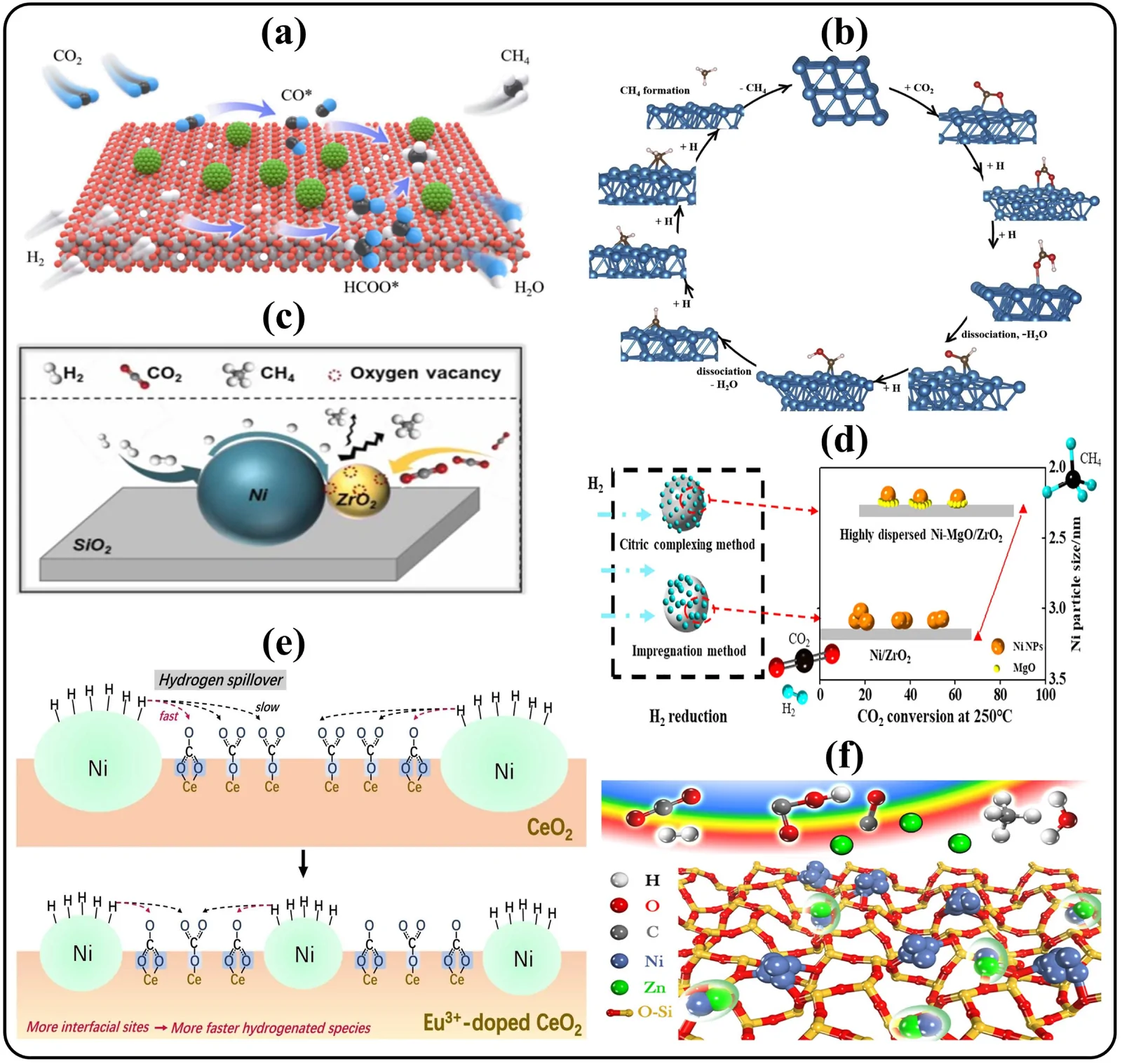
Representative illustrations of Ni-based CO_2_ hydrogenation mechanisms and modification strategies: (a) Two main mechanistic routes for CO_2_ methanation involving *CO and *COOH intermediates. Reproduced with permission from ref 75. Copyright 2024, RSC. (b) Proposed optimal reaction pathway on the Ni(111) surface, highlighting sequential hydrogenation steps leading to CH_4_ formation. Reproduced with permission from ref 76. Copyright 2019, Elsevier. (c) Incorporation of ZrO_2_ promotes the generation of oxygen vacancies, enhancing CO_2_ activation and conversion. Reproduced with permission from ref 78. Copyright 2022, Elsevier. (d) MgO doping improves Ni nanoparticle dispersion and suppresses sintering, thereby increasing catalyst stability. Reproduced with permission from ref 79. Copyright 2019, Elsevier. (e) Eu^3+^ addition strengthens Ni–O–Ce interfacial bonding, boosting CO_2_ methanation activity. Reproduced with permission from ref 84. Copyright 2022, Elsevier. (f) Zn incorporation enhances Ni dispersion and mitigates CO poisoning by reinforcing Ni–Zn–support interactions. Reproduced from ref 85. Copyright 2023, American Chemical Society.

Support effects play a decisive role in steering CO_2_ hydrogenation selectivity. Reducible oxides, such as CeO_2_, ZrO_2_, and La_2_O_3_, enhance oxygen vacancy formation and strengthen metal–support interactions, thereby improving CO_2_ adsorption and accelerating formate hydrogenation toward CH_4_ production. For instance, Ni/AlCeO_3_ achieved 83% CO_2_ conversion at 200 °C owing to Ce^3+^-induced medium-strength basic sites that stabilized *COOH.^77^ As illustrated in Figure 5c, introducing ZrO_2_ into Ni/SiO_2_ generates abundant oxygen vacancies that weaken C = O bonds and facilitate CO_2_ activation, improving low-temperature methanation.^78^ Similarly, Figure 5d shows that Ni/MgO–ZrO_2_ catalysts achieve up to 90% CO_2_ conversion at 250 °C; although MgO is not catalytically active, it restricts Ni particle growth, mitigating sintering and carbon deposition.^79^ Conversely, weakly interacting supports, such as SiO_2_, or elevated temperatures (>450 °C) favor CO desorption via the RWGS pathway, thereby reducing overall CH_4_ selectivity.^80^ In addition to these oxide supports, zeolite-supported Ni catalysts have also been investigated, as their tunable acidity and confinement effects can influence metal dispersion, reaction pathways, and product selectivity.^81–83^

Promoter incorporation provides another lever to fine-tune the selectivity of CO_2_ hydrogenation. Additives modulate metal–support interactions and modify the electronic structure of Ni sites, favoring either CO or CH_4_ formation. For example, Eu^3+^ doping in Ni–CeO_2_ increased Ce–O–Ni interfacial density and strengthened Ni–CeO_2_ coupling, achieving complete CO_2_ conversion to CH_4_ at 210 °C (Figure 5e).^84^ Similarly, the addition of Zn to Ni/SiO_2_ enhanced bimetallic Ni–Zn interactions, suppressing CO generation and promoting CH_4_ formation through facilitated CO hydrogenation (Figure 5f).^85^ Beyond these examples, Co, Ru, and other rare-earth promoters intensify Ni–support synergy and accelerate H_2_ activation; for instance, bimetallic Ni–Co/MgO and Ru_1_Ni/CeO_2_ systems promote tandem CO dissociation and hydrogenation, enhancing CH_4_ yield while suppressing CO formation.^86,87^

Overall, Ni-based systems serve as dynamic catalytic platforms in which CO vs CH_4_ selectivity is governed by the interplay between kinetic descriptors (CO binding strength and hydrogen coverage) and structural factors such as metal–support interactions. Strengthening H_2_ activation and stabilizing formate species favor complete methanation, while weaker Ni–support coupling or excessive temperatures shift selectivity toward CO. Rational control over Ni particle size, promoter chemistry, and support reducibility thus provides a tunable strategy to achieve selective CO_2_-to-CH_4_ or CO conversion under industrially relevant conditions (e.g., 250–400 °C, 10–30 bar, H_2_/CO_2_ ≈ 3–4).^88^ As summarized in Table 1, recent Ni-based catalysts show excellent low-temperature activity and tunable selectivity. Supports such as CeO_2_, ZrO_2_, and La_2_O_3_ promote nearly complete CH_4_ selectivity even below 300 °C, whereas bimetallic systems including Ni–Ga/CeO_2_ and Ni–In/MCM-41 achieve comparable conversions through enhanced metal–support synergy. At higher temperatures, carbon-supported Ni catalysts, e.g., Ni/graphene nanoplatelet (Gnp), favor partial hydrogenation and limited C_2_^+^ formation, illustrating the structural versatility of Ni platforms across reaction regimes.

**Table 1:** Catalytic Performance of Ni-Based Catalysts for CO_2_ Hydrogenation, Comparing CO_2_ Conversion and Product Selectivity under Varying Reaction Conditions

	Reaction conditions					Selectivity (%)			
Catalyst	H_2_: CO_2_	GHSV (mL h^–1^gcat ^–1^)	T (°C)	P (MPa)	CO_2_ conv. (%)	CO	CH_4_	C_2_^+^	C_2_^+^
Ni/Ga_4_–CeO_2_	4	24,000	400	0.1	81.4	0.9	99.1	NA	Zhang et al.^89^
NiGa_2_/CeO_2_					17.9	99.7	0.3		
Ni@MCM-41	4	30,000	400	1.0	∼89	NA	100	NA	Zhang et al.^90^
NiIn@MCM-41	4	30,000	300	1.0	-	97	3		
Ni–Ce nanorod	3	10,800	320	0.1	39.5	1.5	95.9	2.6	Gupta et al.^91^
Ni–Ce nanopolyhedra					28.3	1.6	96.0	1.4	
Ni/Gnp	5	4,850	500	NA	60	83	17	NA	Ebrahim et al.^92^
Ni/CeO_2_–K	4	13,500	500	NA	48.6	93.8	6.4	NA	Zhang et al.^93^
Ni/La_2_O_3_	4	7,200	400	0.1	77	3	97	NA	Zhong et al.^94^
Ni/ZrO_2_	4	12,000	230	1.0	84	1.4	98.6	NA	Ye et al.^95^
Ni/SiO_2_			300		88.4	0.7	99.3		
			240		11.5	4.6	95.0		
Ni/CeO_2_	4	45,000	300	NA	85.7	14	86	7.8	Ullah et al.^96^
NiP/SiO_2_	4	43,200	600	0.1	36.8	100	NA	NA	Bao et al.^97^
NiIn/SBA-15	4	24,000	400	0.1	29	99	1	NA	Zhang et al.^98^
Ni/ZnO	4	36,000	400	1.0	21	99.3	0.7	NA	Lin et al.^99^
Ni–Pt/SiO_2_ nanorod	3	9,600	400	0.1	59	4	96	NA	Huang et al.^100^
mNiSiO_3_	3	8,000	400	0.1	22.9	19.1	80.9	NA	Liu et al.^101^
mNiSiO_3_/GO					13.7	57.4	42.6		
Ni/Cu	4	60,000	400	NA	-	∼90	∼10	NA	Deng et al.^102^
Ni/Mg–Al_2_O_4_	4	-	400	NA	59.9	5	95	NA	Feng et al.^103^
Ni-in-Cu	3	-	550	0.1	26.0	>99.9	NA	NA	Wang et al.^104^

### 3.2 Fe-Based and Tandem Systems for C_2_^+^ Hydrocarbons

Fe-based catalysts are among the most versatile systems for CO_2_ hydrogenation, combining RWGS and FTS functionalities within a single catalytic system.^6,24,105^ This dual behavior enables CO_2_ to first reduce to CO via RWGS, followed by C–C coupling and chain growth over iron carbides (primarily χ-Fe_5_C_2_), providing a sustainable route to C_2_^+^ hydrocarbons.^27,106^ The reaction proceeds through a dynamic redox–carburization cycle in which Fe_3_O_4_ catalyzes RWGS, while Fe_5_C_2_ governs C–C bond formation.^7,24^ Operando spectroscopic analyses (e.g., X-ray diffraction (XRD), X-ray absorption (XAS), and Raman) reveal that the coexistence and transformation of these carbides dictate product selectivity: moderate activity favors C_2_–C_4_ olefins, whereas excessive carburization drives overhydrogenation to CH_4_.^6,24^ As shown in Figure 6a, Fe_5_C_2_ is dominant in the upstream zones of Fe-CP (coprecipitated Fe_2_O_3_) and Fe-TD (thermally decomposed Fe_2_O_3_) catalysts, while Fe_3_O_4_ becomes prevalent toward the outlet, consistent with increasing H_2_O partial pressure.^107^ Although Fe-TD contains a higher overall Fe_5_C_2_ fraction than Fe-CP, its intrinsic CO_2_ conversion and hydrocarbon formation rates are lower, indicating that the spatial profile and structural origin of Fe_5_C_2_, influenced by precursor type and carburization pathway, critically determine catalytic activity and reconstruction of carbides.^107^

**Figure 6:**
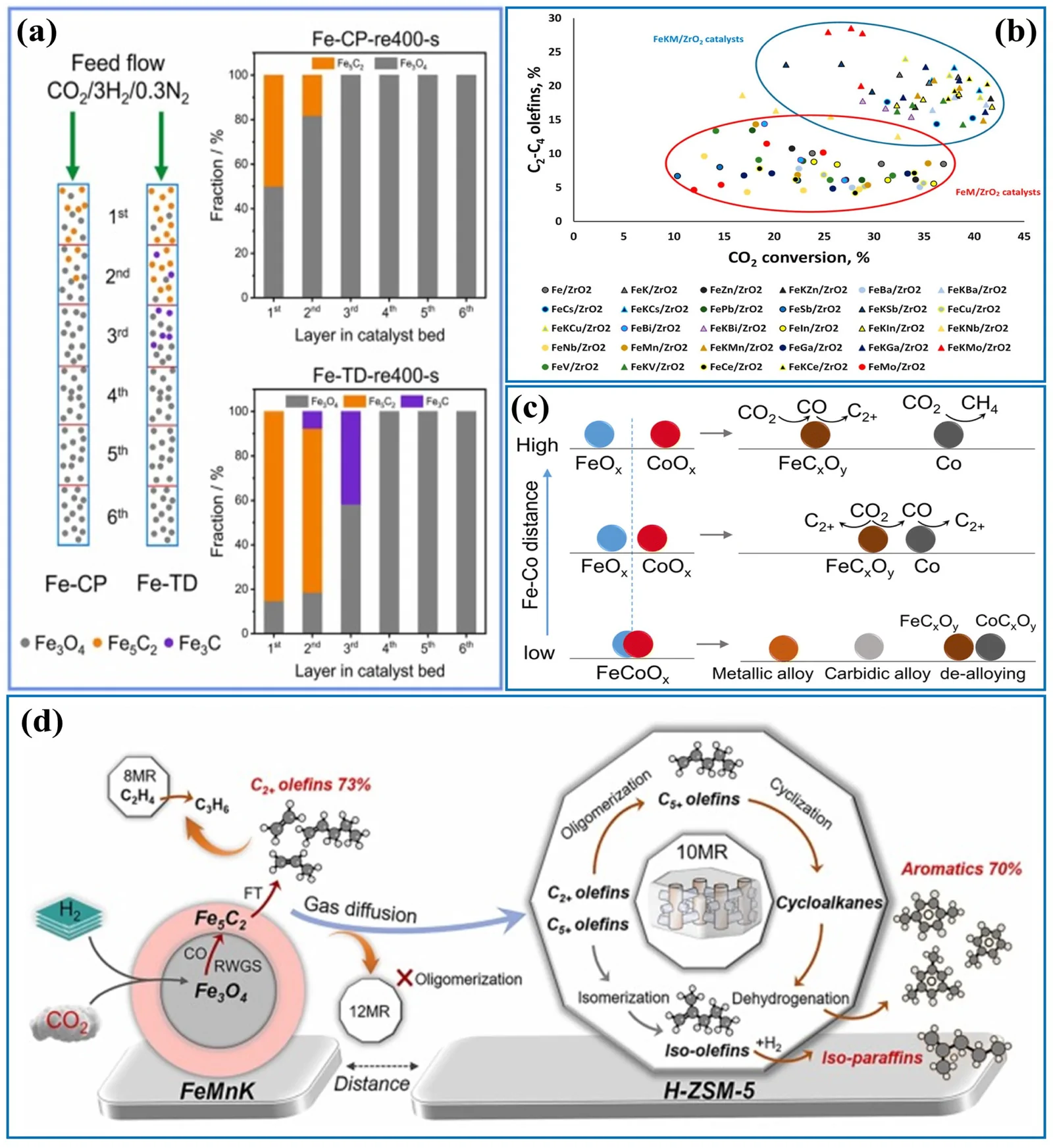
(a) Spatially resolved distribution of Fe-containing phases in Fe-CP and Fe-TD catalysts after reduction at 400 °C, as determined by Mössbauer spectroscopy. Reproduced with permission from ref 107. Copyright 2023, Elsevier. (b) Correlation between CO_2_ conversion and light olefin selectivity over Fe/ZrO_2_-based catalysts at 350 °C (H_2_/CO = 3, WHSV = 4.67–18.19 L g^–1^ h^–1^, *P* = 10 bar, TOS = 50 h). Reproduced from ref 108. Copyright 2022, American Chemical Society. (c) Schematic illustration of alloying–dealloying dynamics in Fe–Co bimetallic catalysts during activation and CO_2_ hydrogenation. Reproduced from ref 6. Available under a CC-BY 3.0 license. Copyright 2025, RSC. (d) Tandem catalytic mechanism of CO_2_ hydrogenation over FeMnK–zeolite bifunctional catalysts. Reproduced with permission from ref 114. Copyright 2023, Elsevier.

Promoters and supports are key to tuning this delicate balance. Alkalis (K, Rb, Cs) enhance surface basicity and stabilize Fe carbides, while transition-metal promoters (Mn, Cu, Zn, and Ga) improve carbide dispersion and carburization, thereby steering selectivity toward longer chains.^23,108–110^ The synergy of alkali and transition metals is frequently reported. For instance, Barrios et al.^108^ demonstrated that pairing K with Ce, Cu, Ga, or Mo on Fe/ZrO_2_ markedly increased C_2_–C_4_ olefin selectivity (Figure 6b), owing to enhanced Fe dispersion, deeper carburization, and stronger basicity. Similarly, oxygen-vacant supports, such as ZrO_2_, further facilitate CO_2_ activation and Fe-carbide stabilization, complementing promoter effects and enhancing chain-growth efficiency.^111–113^

Beyond monometallic systems, tandem and bimetallic architectures expand the product spectrum. Coupling Fe_5_C_2_ with acidic or zeolitic domains (e.g., H-ZSM-5) enables in situ oligomerization and aromatization of primary olefins, forming gasoline-range hydrocarbons while maintaining high CO_2_ conversion.^25,114^ Bifunctional Fe–zeolite catalysts exemplify the tandem approach to CO_2_ hydrogenation, in which metal and acid sites cooperate to promote carbon-chain growth. As illustrated in Figure 6d, the FeMnK catalyst forms a Fe_3_O_4_@Fe_5_C_2_ core–shell structure, in which Fe_3_O_4_ promotes CO_2_-to-CO via the RWGS reaction, and Fe_5_C_2_ promotes CO hydrogenation to C_2_^+^ olefins with ∼73% selectivity.^114^ These olefins subsequently diffuse into the 10-membered-ring (10MR) micropores of H-ZSM-5, where Brønsted acid sites catalyze oligomerization, cyclization, and isomerization, producing hydrocarbons with carbon numbers ranging from C_5_ to C_11_. The shape-selective nature of 10MR channels favors efficient olefin upgrading compared to smaller (8MR) or larger (12MR) frameworks.

Maintaining an optimal Fe–zeolite proximity is crucial, as alkali migration can neutralize acid sites. Fe–Cu and Fe–Zn tandems exploit interfacial synergy between carbide and metallic sites to balance CO formation and hydrogenation kinetics.^6^ As illustrated in Figure 6c, Fe–Co systems further refine this concept: depending on the Fe/Co ratio and reaction atmosphere, catalysts can evolve into alloys, segregated, or mixed-phase structures.^6^ A moderate Fe–Co proximity effectively harmonizes RWGS, CH_*x*_ coupling, and olefin hydrogenation, allowing tailored control over the hydrocarbon product spectrum. As summarized in Table 2, Fe-, Co-, and tandem Fe–Co-based catalysts exhibit distinct CO_2_ hydrogenation behaviors depending on promoter type and support. Alkali- and transition-metal-promoted Fe systems, such as K–Fe and K–Sr/Fe_3_O_4_, deliver high CO_2_ conversion with enhanced C_2_–C_4_ olefin and C_5_^+^ hydrocarbon selectivity due to improved carburization and CO_2_ activation.^115,116^ Bimetallic Fe–Co catalysts further balance RWGS and FTS functions, enabling efficient C–C coupling and suppressing overhydrogenation to CH_4_.^117,118^ These studies highlight how promoter–support synergy and Fe–Co interactions can selectively guide CO_2_ hydrogenation toward light olefins and gasoline-range hydrocarbons.

**Table 2:** Catalytic Performance of Representative Fe- and Fe–Co-Based Catalysts for CO_2_/CO Hydrogenation to Hydrocarbons

	Reaction conditions				CO_2_ conv. (%)	Selectivity (%)					
Catalyst	H_2_: CO_2_	GHSV (mL h^–1^gcat ^–1^)	T (°C)	P (MPa)	CO_2_ conv. (%)	CO	CH_4_	C_2_^0^–C_4_^0^	C_2_^=^–C_4_^=^	C_5_^+^	C_5_^+^
Na/Fe_2_O_3_	3	10,000	320	1.5	38.8	10.5	46.6	5.4	33.9	14.0	Zhang et al.^119^
Na/Fe_4_N					29.8	31.4	32.9	5.4	41.0	20.8	
K/Fe	3	586	300	1.5	36.9	13.9	13.9	6.9^b^	35.3	30.0	Krausser et al.^115^
K–Sr/Fe_3_O_4_	3	5,000	340	2.5	45.4	11.6	10.4	18.7^d^	NA	70.9^e^	Zhong et al.^116^
CoFe	3	7,337	300	0.2	20.8	77.3	11.5	11.2^c^	NA	NA	Yan et al.^120^
Cu/Fe_3_O_4_/Mo_2_CT_*x*_	3^a^	12,000	320	2.5	78.5^f^	16.4^g^	48.7	36.1^d^	NA	15.2^e^	Zhou et al.^121^
CoFe	3	8,000	320	2	48.0	6.1	NA	64.9^c^	NA	NA	Liu et al.^122^
Fe@FeO_*x*_-FeC_*y*_–Mn-K	3	5,000	320	1.5	40.5	5.5	7.9	32.1^d^	NA	60^e^	Liu et al.^123^
Fe@FeO_*x*_-FeC_*y*_-K	3	10,000	300	1	38.1	10.5	27.3	37.4^d^	NA	35.3^e^	
FeCo 3-BCN	3^a^	-	230	0.18	2.2^f^	78.7^g^	56.4	36.6^b^	NA	7.0	Hao et al.^118^
FeCo	1^a^	3000	250	6	82.8^f^	43^g^	10.1	62.6^c^	NA	NA	Gong et al.^117^
Co–Fe_5_C_2_/TiC	3^a^	-	350	0.5	NA	∼30^g^	41	32^d^	NA	27^e^	Jiang et al.^124^
AlFe	3	6,000	300	NA	18.1	24.9	29.8	44.3^b^	NA	1.0	Rosa-Priego et al.^125^
Fe/FeOx/Al_2_O_3_–MgO	4	-	275	0.18	50.1	20.3	47.1	46.6^b^	NA	6.3	Li et al.^126^
Fe_5_C_2_	3	7200	320	3	49.8	2.8	46.1	35.7	3.8	11.6	Liu et al.^20^
K–Fe_5_C_2_					46.8	9.7	22.7	8.6	38.7	19.5	
CoFe	2^a^	-	280	0.2	33.6^f^	26.9^g^	12.1	11.5	30.7	33.7	Fadlalla et al.^127^
NiFe					38.1^f^	35.2^g^	30.4	20.6	20.7	11.1	
Co–Ni–Fe					41.9^f^	35.0^g^	32.5	18.4	21.2	11.9	
Na-CoFe_2_O_4_/CNT	3	3,600	340	0.1	34.4	18.6	14.8	5.5	38.8	40.9	Kim et al.^128^

a H_2_/CO.

b C_2_–C_4_.

c C_2_^+^.

d C_2_–C_3_.

e C_4_^+^.

f CO conversion.

g CO_2_ selectivity.

Overall, Fe-based and tandem catalytic systems exemplify how phase evolution, promoter–support interactions, and intermetallic coupling cooperatively govern the selectivity of CO_2_ hydrogenation. Rational control over Fe-carbide dispersion, defect chemistry, and alloy or bifunctional interfaces enables precise tuning of the C_1_ and C_2_^+^ product ratios. These insights establish a robust framework for designing next-generation catalysts that can efficiently convert CO_2_ into higher hydrocarbons and olefins under industrially relevant conditions.

### 3.3 Cu-Based and Oxide-Supported Systems for CH_3_OH Synthesis

Cu-based catalysts are the most extensively studied and industrially relevant systems for CO_2_ hydrogenation to methanol, owing to their ability to selectively stabilize oxygenated intermediates and suppress deep hydrogenation.^1,129,130^ In contrast to Ni-based catalysts favoring methane and Fe-based systems promoting C_2_^+^ hydrocarbons, Cu-based systems preferentially drive the formation of methanol through a balanced hydrogenation pathway. The reaction proceeds via the exothermic hydrogenation of CO_2_ (Reaction 23), in competition with the endothermic reverse water–gas shift (RWGS) reaction, which significantly influences selectivity under reaction conditions.^129^

23 $$CO_{2}+3H_{2}⇋CH_{3}OH+H_{2}O\;\Delta H=-49.5\;\mathrm{kJ}/\mathrm{mol}$$

The selectivity toward methanol is strongly dependent on operating conditions. Lower temperatures and higher pressures thermodynamically favor methanol formation, whereas higher temperatures enhance CO_2_ activation but promote RWGS and byproduct formation such as CO and CH_4_.^129,131^ Additionally, water formed during the reaction can inhibit catalytic activity and contribute to deactivation, highlighting the need for catalysts with high stability and resistance to poisoning.^132,133^

The Cu/ZnO/Al_2_O_3_ (CZA) system remains the industrial benchmark for methanol synthesis, originally developed for syngas conversion and later adapted for CO_2_ hydrogenation.^134,135^ The superior performance of CZA catalysts arises from the strong interaction between Cu and ZnO, where Cu provides active hydrogenation sites, and ZnO enhances the dispersion and stability of Cu nanoparticles.^136,137^ The Cu–ZnO interface is widely regarded as the active site, with Zn species dynamically interacting with Cu under reaction conditions to form highly active interfacial structures.^138,139^ The morphology and exposed facets of ZnO significantly influence catalytic performance. For instance, ZnO surfaces with higher oxygen vacancy concentrations enhance CO_2_ activation and improve methanol synthesis rates.^140,141^ Various structural designs, including core–shell Cu–ZnO systems, nanoalloys, and graphitic ZnO structures, have been developed to further improve activity and stability.^138,139,142,143^

To enhance performance, Cu-based catalysts are often modified with various supports and promoters. Al_2_O_3_ improves structural stability, while ZrO_2_ enhances CO_2_ adsorption and provides resistance to water poisoning.^144–146^ The introduction of Zr and other oxides increases the concentration of oxygen vacancies and strengthens metal–support interactions, thereby improving catalytic activity and methanol selectivity.^129^ Promoters such as Ga, Mn, and In further modify the electronic and structural properties of Cu-based systems. For example, Ga_2_O_3_ promotes the formation of Cu^+^ species and enhances hydrogenation activity, whereas ZrO_2_-based systems exhibit improved water tolerance and higher CO_2_ conversion.^147–150^ These modifications demonstrate that catalyst performance is strongly dependent on the interplay between active metal sites and support properties.

Beyond conventional Cu/ZnO systems, bimetallic and alloy catalysts have been widely explored to improve catalytic efficiency. Systems such as Pd–Cu, Ni–Ga, Au–Cu, and Pd–Zn exhibit enhanced activity due to synergistic interactions between metals.^150–152^ In particular, PdZn alloys have been identified as highly active phases, where smaller alloy particle sizes and optimized dispersion lead to improved methanol selectivity.^153,154^ In these systems, a bifunctional mechanism is often observed, where the metal component activates H_2_, and the oxide support facilitates CO_2_ adsorption and transformation into intermediate species such as formates.^129^ This cooperative interaction enhances reaction rates and selectivity toward methanol. The catalytic trends are summarized in Table 3. Cu-based catalysts, particularly Cu–ZnO–Al_2_O_3_, show high methanol selectivity under moderate conditions, confirming their industrial relevance. Modified Cu-based and bimetallic systems exhibit tunable performance depending on support and reaction parameters. Notably, oxide-based catalysts such as In_2_O_3_ and ZnO–ZrO_2_ achieve very high methanol selectivity, highlighting the key role of metal–oxide interfaces and oxygen vacancies in directing CO_2_ hydrogenation toward methanol.

**Table 3:** CO_2_ Hydrogenation to CH_3_OH Using Transition Metal- and Oxide-Based Catalysts

Catalyst	Reaction conditions					
Catalyst	T (°C)	GHSV (mL h^–1^gcat ^–1^)	P (MPa)	P (MPa)	P (MPa)	P (MPa)
*Transition metal-based catalysts*						
Cu–ZnO–ZrO_2_	200	7,800	3.9	3.9	70.0	Portha et al.^159^
Cu–ZnO–ZrO_2_	280	10,000	5	21.0	34.0	Angelo et al.^160^
Cu/ZrO_2_	220	-	0.1	0.53	19.8	Kattel et al.^161^
Cu/ZrO_2_	280	-	3	12.0	32.0	Witoon et al.^162^
Cu–ZnO–Al_2_O_3_	280	10,000	4.2	65.3	91.9	Gaikwad et al.^163^
Pd/ZnO	250	-	2	10.7	60.0	Bahruji et al.^154^
Au/ZnO	240	-	0.5	1.0	70.0	Hartadi et al.^164^
Pd–Cu/SiO_2_	300	3,600	4.1	6.6	34.0	Jiang et al.^151^
Au/Cu–ZnO–Al_2_O_3_	260	7,000 – 12,200	1 – 6	28.0	55.0	Pasupulety et al.^152^
Cu–ZnO–ZrO_2_	240	10,000	5	23.0	33.0	Angelo et al.^165^
Cu–ZnO–ZrO_2_	240	3,600	3	12.1	54.1	Li et al.^166^
Au/ZnO	220	-	0.5	0.2	56.2	Hartadi et al.^167^
*Oxide-based catalyst*						
In: Pd (2:1)/SiO_2_	300	-	5	-	61.0	Snider et al.^168^
Cu–In–Zr–O	250	18,000	2.5	1.48	79.7	Yao et al.^169^
Ga_0.4_In_2–x_O_3_	320	-	3	12.5	26.4	Akkharaphatthawon et al.^170^
In_5_/ZrO_2_	280	24,000	5	-	60.0	Chen et al.^171^
Pd/In_2_O_3_	300	>21,000	5	>20	>70	Rui et al.^172^
ZnO–ZrO_2_	315	24,000	5	>10	91.0	Wang et al.^173^
In_2_O_3_/ZrO_2_	300	16,000	5	5.2	99.8	Martin et al.^37^
In_2_O_3_	330	15,000	4	7.1	39.7	Sun et al.^174^

Mechanistically, methanol synthesis over Cu-based catalysts is generally described by the formate pathway, involving the sequential hydrogenation of CO_2_ to formate and methoxy intermediates. However, the exact reaction pathway remains debated, with alternative RWGS-mediated routes also proposed.^17,155^ As illustrated in Figure 7a, methanol formation can proceed via both CO_2_ hydrogenation through formate intermediates and CO hydrogenation via formyl species, highlighting the coexistence of multiple mechanistic routes. In this context, the balance between CO_2_ activation and H_2_ activation remains critical, particularly at metal–oxide interfaces such as Pd/In_2_O_3_, where the proximity between hydrogen activation sites and CO_2_ adsorption sites plays a key role in determining catalytic performance. In such systems, alloying may enhance activity; however, interfacial synergy between distinct active sites is often more important than bulk alloy formation.^37,156^ In addition, oxide-based systems such as In_2_O_3_ introduce oxygen vacancies (Ov) that act as active sites for CO_2_ activation. As shown in Figure 7b, these vacancies stabilize key intermediates such as *COOH, *H_2_COO, and *CH_2_O, facilitating their sequential hydrogenation. The hydrogenation of *CH_2_O has been identified as the rate-determining step, while the vacancy sites are regenerated during the catalytic cycle, enabling sustained activity.^129,157^

**Figure 7:**
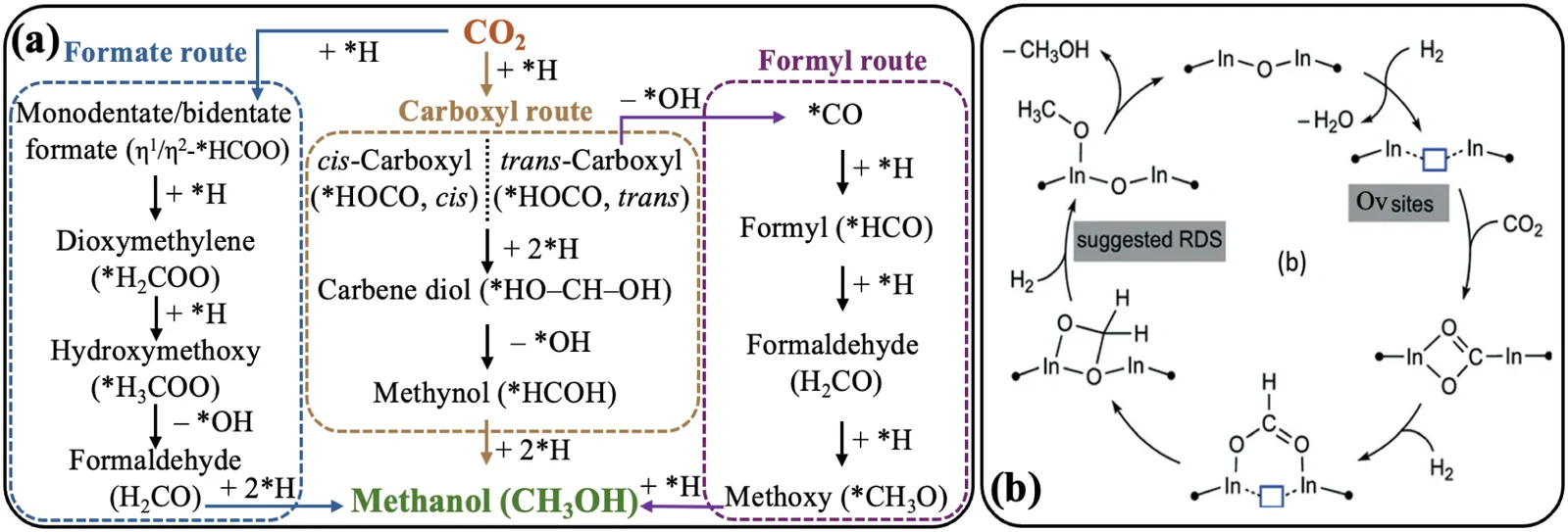
(a) Mechanistic pathways for conversion of CO and CO_2_ to CH_3_OH over Cu. Adapted with permission from ref 158. Copyright 2013, Elsevier. (b) The hydrogenation of CO_2_ to CH_3_OH on Ov sites of In_2_O_3_. Reproduced from ref 157. Copyright 2019, American Chemical Society.

### 3.4 Emerging Catalysts

The search for next-generation catalysts for CO_2_ hydrogenation has expanded beyond conventional metals and oxides to include transition-metal carbides (TMCs), phosphides, and high-entropy materials (HEMs). These emerging classes provide tunable electronic structures, strong metal–support coupling, and improved thermal stability, offering new design pathways for selective C–O bond activation and C–C coupling.

TMCs have emerged as highly tunable catalysts for CO_2_ hydrogenation due to their strong metal–carbon covalency, Pt-like electronic structures, and robust metal–support interactions (MSI), which collectively enable efficient CO_2_ activation and selective hydrogenation.^18^ Their catalytic behavior is strongly governed by atomic ratios, surface terminations, and crystallographic orientation. Adjusting the metal/carbon ratio alters the balance between nondissociative and dissociative CO_2_ activation: stoichiometric δ-MoC(001) primarily yields CO or methanol through the formate pathway, while Mo-rich β-Mo_2_C­(001) promotes complete C–O bond cleavage and CH_4_ formation, though excessive metal exposure can lead to carbon deposition and lower stability.^175^ Density-functional calculations show that Mo-terminated facets possess higher CO_2_ adsorption energies (≈−3.27 eV) and enable complete C–O scission. In contrast, C-terminated surfaces favor molecular adsorption and partial reduction.^175^ Beyond atomic ratios and termination effects, facet-dependent reactivity also governs product distribution in TMC-catalyzed CO_2_ hydrogenation. On χ-Fe_5_C_2_, kinetic analysis reveals that the (510) facet exhibits the highest TOFs for CH_4_, CO, and minor C_2_ products, with methane dominating as temperature increases (Figure 8a). The low C_2_ selectivity is attributed to dense *O coverage, which blocks the 3-fold hollow sites required for C–C coupling, consistent with experimental observations on iron carbides.^176^ Although experiments report comparable CO and C_2_ selectivity, these differences likely arise from coverage and competitive adsorption effects not captured in current calculations. These insights highlight that high-index planes such as χ-Fe_5_C_2_(510) favor direct CO_2_ dissociation and fast hydrogenation to CH_4_, whereas lower-index surfaces (e.g., χ-Fe_5_C_2_(111)) preferentially follow *HCOO pathways that promote oxygenate or CO formation.^177^ Experimental evidence from 2D-Mo_2_C nanosheets further confirms that maximizing exposed Mo sites enhances CO_2_ conversion rates by nearly an order of magnitude relative to bulk β-Mo_2_C; this improvement arises from the fully accessible basal planes generated during the controlled defunctionalization of Mo_2_CT_*x*_, as illustrated in the schematic preparation route (Figure 8b).^178^ Overall, tailoring the atomic composition, surface termination, and crystal facets of TMCs provides a powerful means to control the strength of CO_2_ activation and intermediate binding, thereby steering selectivity between CO, CH_4_, and higher hydrocarbons while maintaining catalyst stability.

**Figure 8:**
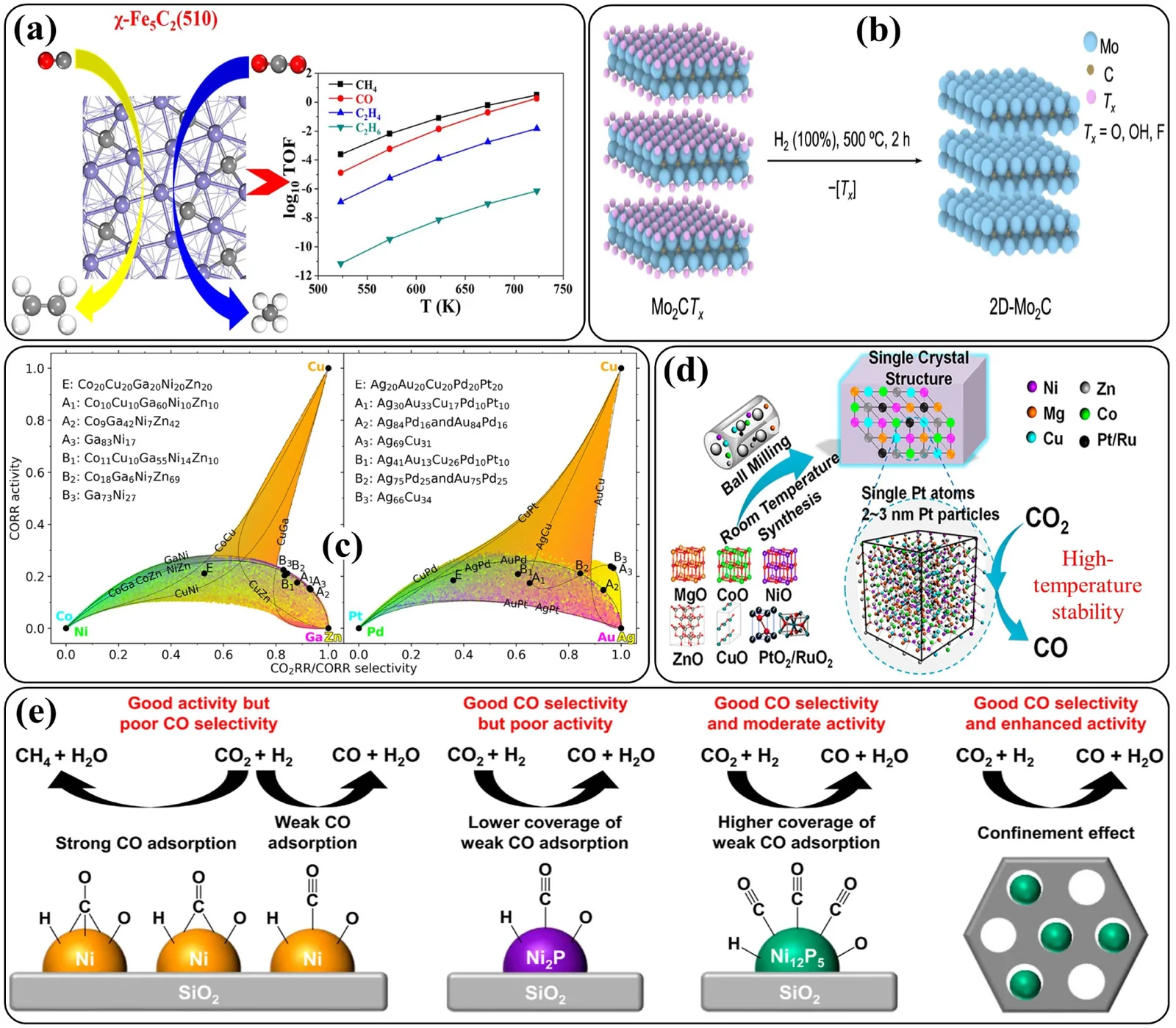
(a) TOF of the major CO_2_ hydrogenation products over χ-Fe_5_C_2_(510) as a function of temperature, reflecting the intrinsic activity of this carbide surface (gray: C; red: O; white: H; blue: Fe of iron oxide). Reproduced from ref 177. Copyright 2022, American Chemical Society. (b) schematic pathway showing the conversion of Mo_2_CT_X_ precursor into 2D-Mo_2_C. Reproduced from ref 178. Available under a CC-BY 4.0 license. Copyright 2021, Springer Nature. (c) Plots illustrating the relationship between CO_2_RR/CORR selectivity and CORR activity for CoCuGaNiZn (left) and AgAuCuPdPt (right), highlighting the range of performance achievable within each alloy composition space. Reproduced from ref 182. Copyright 2020, American Chemical Society. (d) Mechanochemical preparation of Pt/Ru-incorporated (NiMgCuZnCo)O entropy-stabilized metal oxide solid solutions. Reproduced from ref 184. Copyright 2019, American Chemical Society. (e) CO_2_ hydrogenation pathways on Ni/SiO_2_, Ni_2_P/SiO_2_, Ni_12_P_5_/SiO_2_, and Ni_12_P_5_@SBA-15. Reproduced from ref 97. Copyright 2023, American Chemical Society.

High-entropy materials have emerged as promising catalysts for CO_2_ conversion because their multicomponent lattices generate diverse active-site environments that can tune CO_2_ adsorption, intermediate binding, and hydrogen activation in ways that traditional catalysts cannot.^179–181^ Their high configurational entropy stabilizes disordered metal or oxide frameworks under reaction conditions, mitigating deactivation pathways and enabling more effective C–O bond activation. In high-entropy alloys (HEAs), the broad distribution of surface ensembles creates a continuously variable reaction landscape where catalytic behavior shifts systematically with composition. Using Gaussian process regression to explore this compositional space, Pedersen et al.^182^ constructed selectivity–activity maps that correlate predicted adsorption energies with CO_2_RR/CORR performance. These maps revealed that adjusting elemental ratios allowed movement across distinct catalytic regimes, with equimolar HEAs occupying regions associated with strongly reducing surfaces that stabilize more hydrogenated carbon intermediates (Figure 8c). This highlights how the compositional degrees of freedom unique to HEAs can guide catalyst optimization beyond conventional alloy design. Complementary experiments confirm that entropy-stabilized multimetallic surfaces can suppress hydrogen evolution and promote selective CO formation.^183^ High-entropy oxides (HEOs) further extend this concept: rock-salt (NiMgCoCuZn)­O, synthesized via mechanochemical routes, provides a thermally robust matrix that uniformly accommodates noble-metal species. As illustrated in Figure 8d, mechanical energy and frictional heating alone enable rapid room-temperature crystallization of single-phase (NiMgCoCuZn)­O, while the entropy-stabilized lattice allows Pt or Ru to disperse as nanoclusters or even isolated single atoms through solid-state grinding.^184^ This solvent-free, energy-efficient synthesis produces compositionally complex oxides with a high density of accessible active sites and exceptional structural stability, underscoring the versatility of entropy-driven materials design for CO_2_ hydrogenation. These findings position high-entropy materials as an emerging category of CO_2_ hydrogenation catalysts, in which compositionally complex surfaces, entropy-driven stabilization, and multisite synergies create new opportunities to tune activity and selectivity beyond what is achievable with traditional catalyst families.

Phosphide-based catalysts have recently gained attention as a unique platform for CO_2_ hydrogenation, largely because phosphidation reshapes how metal surfaces interact with CO_2_, H_2_, and CO. During phosphidation, electron density is partially transferred from the transition metal to phosphorus, while phosphorus atoms insert between neighboring metal atoms, increasing their separation and altering the surface electronic structure. These effects weaken the metal’s tendency to hydrogenate *CO. This combination explains why phosphides consistently favor CO production over CH_4_ across different metal phosphides.^97,185,186^ This trend is most clearly illustrated in ruthenium phosphides, which show a dramatic transformation upon phosphidation. Metallic Ru behaves as a methanation catalyst. But after converting part of the surface into Ru_2_P, its product selectivity shifts sharply from CH_4_ to CO. For the Ru–Ru_2_P heterostructure, Ru sites retain sufficient metallic character to activate CO_2_ and H_2_, while adjacent Ru_2_P domains prevent the formation of multibonded CO species that lead to methane.^185^ Operando DRIFTS confirms that *CO is the stable intermediate on Ru–P surfaces, and DFT indicates that the hydrogenation barrier for *CO increased to prevent its reduction to CH_4_.^185^ In other words, Ru_2_P does not merely weaken CO adsorption; it fundamentally alters the reaction landscape so that CO is the only kinetically accessible product. This mechanistic pivot forms the conceptual backbone of the phosphide strategy.

The behavior of Ni-phosphides follows the same mechanistic logic, as shown in Figure 8e. On Ni/SiO_2_, CO_2_ dissociates to form strongly bound multibonded CO species (bridge- and 3-fold-CO), which readily hydrogenate to CH_4_, explaining the poor CO selectivity.^97^ After phosphating, these multibonded configurations are no longer accessible because P atoms spatially isolate Ni sites. Only weak, linearly bonded *CO is observed on Ni_12_P_5_ and Ni_2_P, and this species desorbs directly as CO. The higher coverage of linear CO on Ni_12_P_5_/SiO_2_ relative to Ni_2_P/SiO_2_ reflects its stronger metallic character and accounts for its superior RWGS activity. Confining Ni_12_P_5_ nanoparticles within the pores of SBA-15 further enhances dispersion and prevents sintering, yielding higher CO_2_ conversion while maintaining near-unity CO selectivity.^97^

The same principles extend to Mo-based phosphides. Although Mo–P systems generally show lower intrinsic activity than their Ru- and Ni-based counterparts, they share the hallmark behavior of CH_4_ suppression and remain remarkably stable in high temperatures.^186^ Their performance further supports the idea that phosphidation is a tunable strategy applicable across multiple metals, each gaining CO selectivity through the same underlying geometric and electronic modifications. Across Ru, Ni, and Mo systems, a clear unifying picture emerges that phosphides do not achieve CO selectivity by simply weakening CO binding. Instead, they inhibit the kinetic pathways required for CO hydrogenation by increasing activation barriers or destabilizing key intermediates, thereby limiting the deep-reduction route to CH_4_.^97,185,186^ By isolating metal sites and altering surface electron density, TMPs direct the reaction exclusively toward CO formation while remaining durable under the harsh thermal conditions required for RWGS. This intrinsic CO-selective behavior positions transition-metal phosphides among the most consistent and mechanistically robust families of catalysts for CO_2_-to-CO conversion.

Table 4 provides a comparative summary of representative carbide, HEM, and phosphide catalysts reported for CO_2_ hydrogenation. Carbide-based systems generally exhibit moderate CO_2_ conversion with diverse product distributions, reflecting their flexible metal–carbon chemistry. Phosphide catalysts consistently show near-unity CO selectivity, aligning with mechanistic evidence that phosphidation suppresses deeper hydrogenation pathways. High-entropy materials also exhibit strong CO selectivity and thermal stability, driven by multimetal synergy and lattice distortion. However, the number of reported phosphide and HEM catalysts for CO_2_ hydrogenation remains limited, indicating that these promising classes are still in the early stages of development compared to the more established carbide literature. Furthermore, key catalyst characteristics and selectivity trends are summarized in Table 5. The table highlights the strong dependence of product selectivity on reaction intermediates, pathway energetics, and catalyst design.

**Table 4:** Catalytic Performance of TEC, HEM, and Phosphide Catalysts for CO_2_ Hydrogenation

	Reaction conditions					Selectivity (%)					
Catalyst	H_2_: CO_2_	GHSV (mL h^–1^gcat ^–1^)	T (°C)	P (MPa)	CO_2_ conv. (%)	CO	CH_4_	C_2_^0^–C_4_^0^	C_2_^=^–C_4_^=^	C_5_^+^	C_5_^+^
*Carbide-based catalyst*											
NaCoFe carbides	3	4,000	320	3	43.8	7.1	16.1	53.7^a^	NA	30.2	Han et al.^187^
Fe_7_C_3_	3	6,000	340	2	41.5	12.4	10.5	37.4^a^	NA	39.6	Qian et al.^188^
FeK-activated carbon	3	4,800	320	2	29.3	28.1	10.5	6.6	24.1	58.8	Huang et al.^189^
Na–Fe_3_O_4_/Fe_5_C_2_	3	4,000	340	3	45.6	6.1	33.0	33.6^a^	NA	27.2	Najari et al.^190^
W_*x*_C	3	7,500	350	2.1	13.9	94.6	5	0.4^b^	NA	NA	Juneau et al.^191^
Fe_2_N@C	4	36,000	250	1	33.7	15.8	46.0	15.4	31.0	7.5	Zhao et al.^192^
α-MoC_1–*x*_	2.7	40,000	300	2	19.8	79.2	16.3	4.4^b^	NA	NA	Baddour et al.^193^
Na–CoFe_2_O_4_/CNT	3	3,600	340	1	34.4	18.6	14.8	5.5	38.8	40.9	Kim et al.^128^
*High-entropy materials*											
FeNa/FeCoCu ZnMnAl_2_O_4_	3	1,650	280	2.5	40.0	5.5	31.3	8.6	39.3	20.9	Ni et al.^194^
MnFeCoNiCuO_*x*_	3	40,000	500	0.1	43.5	100	NA	NA	NA	NA	Chou et al.^195^
MnCoNiCuZnO_*x*_					38.3	100					
NiCoCuZnMgCeO_*x*_	4	10,800	500	NA	48	99	1	NA	NA	NA	Cortazar et al.^196^
PtPdIrRhRu-TiO_2_	5	60,000	450	NA	62	15.1	84.9	NA	NA	NA	Dai et al.^197^
CNF@TiO_2_–HEA	4	120,000	600	0.1	52	97.8	2.2	NA	NA	NA	Ahn et al.^198^
CNF@ZrO_2_–HEA					41.3	100	NA				
*Phosphide-based catalyst*											
Ru–Ru_2_P/SiO_2_	4	43,200	500	NA	35.8	100	NA	NA	NA	NA	Bao et al.^185^
RuP	1	30,000	400	0.1	15.4	64.9	35.1	NA	NA	NA	Wu et al.^199^
Ni_12_P_5_/SiO_2_	4	-	600	NA	36.8	100	NA	NA	NA	NA	Bao et al.^97^
Ni_12_P_5_–Al_2_O_3_	4	12,000	650	0.1	61	58	40	NA	NA	NA	Zhang et al.^200^
Ni_2_P-SiO_2_					58	84	6				

a C_2_–C_4_.

b C_2_^+^.

**Table 5:** Comparative Summary of Catalyst Systems for CO_2_ Hydrogenation

Catalyst system	Key intermediates	Dominant pathway	Selectivity trend	Key limitation	Sustainability implications	ref.
Ni-based	*CO, *CH_*x*_, *C, and *HCOO	RWGS with dissociative methanation	Strong CH_4_ selectivity due to efficient CO dissociation and rapid hydrogenation.	Ni sintering at elevated temperatures.	Highly exothermic reaction requiring effective thermal management.	Ye et al.;^75^ Zhang et al.;^10^ Tommasi et al.;^201^ Zhang et al.;^16^ Molinet et al.^202^
			Associative (formate) pathway is generally less favored.	Limited control over overhydrogenation.	Suitable for synthetic natural gas (SNG) production but limited for selective carbon utilization due to poor C–C coupling.	
Fe-based	*CO, *C, and surface carbide (Fe_3_C/χ-Fe_5_C_2_)	RWGS with Fischer–Tropsch synthesis (FTS)	Selective toward C_2_^+^ hydrocarbons (olefins/paraffins), with product distribution governed by carbide phase and hydrogenation activity	Phase instability	Enables carbon chain growth and higher-value products, but separation and recycle penalties increase process complexity and energy demand.	Jiang et al.;^23^ Krausser et al.;^24^ Zhu et al.;^6^ Liu et al.^203^
				Water sensitivity		
				Methane coproduction		
				Broad product distribution		
Cu-based	*HCOO, *CH_3_O, *CO, and *H_2_COO	Associative hydrogenation (formate pathway)	High CH_3_OH selectivity due to stabilization of formate and methoxy intermediates at Cu–oxide interfaces.	Cu sintering under reaction conditions.	Favorable for methanol production.	Yang et al.;^4^ Sharma et al.;^129^ Guo et al.;^204^ Ye et al.^1^
			CO hydrogenation pathway is less dominant.	Sensitivity to water.	Reducing separation costs.	
				Limited activity at higher temperatures due to thermodynamic constraints.	Compatible with e-fuel synthesis but dependent on stable Cu–support interactions.	
Emerging (carbides, phosphides, HEOs)	*CO, *C, *CH_*x*_, *HCOO, defect/vacancy-stabilized intermediates	Mixed pathways: RWGS and hydrogenation (formate or carbide-like routes), depending on electronic structure and defect chemistry.	Tunable selectivity ranging from CO and CH_4_ to CH_3_OH and C_2_^+^ hydrocarbons, governed by metal–carbon/phosphorus interactions and defect sites.	Limited long-term stability data.	Expands catalyst design space through electronic and defect engineering.	Wang et al.;^18^ Prats et al.;^205^ Li et al.;^206^ Sharma et al.;^207^ Ma et al.;^181^ Sun and Dai^208^
				Surface restructuring under reaction conditions.	Potential for improved stability and resistance to sintering.	
				Complexity in synthesis and reproducibility.	Scalability and durability remain key challenges.	

### 3.5 Section Summary and Design Implications

The performance of CO_2_ hydrogenation catalysts can be rationalized through how effectively they regulate key mechanistic descriptors identified in Section 2. Ni-based systems illustrate the impact of strong hydrogenation activity and CO binding, which often favor methane, whereas Cu-based and oxide-supported systems stabilize formate and methoxy intermediates, promoting methanol synthesis. Fe-based and tandem catalysts enable C–C coupling through carbide formation and multifunctional interfaces, albeit with increased sensitivity to phase stability and water. Emerging materials, including carbides, phosphides, and multication oxides, expand the design space by enabling new combinations of redox behavior and defect structures. However, across all systems, sustained selectivity depends not only on intrinsic activity but on the stability of active phases under operating conditions. These insights highlight the need for descriptor-guided and stability-aware catalyst design, providing a bridge toward data-driven optimization.

## 4 Operando and AI/ML Approaches

### 4.1 Operando Spectroscopy as Mechanistic Insights

By enabling researchers to observe catalytic structures and intermediates in their operational state, in situ/operando spectroscopy has fundamentally changed catalyst development for CO_2_ hydrogenation. The temperature–pressure gap between ex situ characterization and real reaction environments, in which metal oxidation states, surface defects, and active-site ensembles continually change under CO_2_/H_2_ mixtures, is addressed using operando methods.^209,210^ These techniques provide mechanistic insights that guide the rational development of catalysts by observing the evolution of structures and surface intermediates in the working state. The complementary insights obtained from Raman, XAS, FTIR, and steady-state isotopic transient kinetic analysis (SSITKA) contribute to a mechanism-centered understanding of catalytic processes, thereby informing catalyst design strategies.^211^

By observing phase transitions, oxygen-vacancy development, and lattice distortions in real time, Operando/in situ Raman spectroscopy is a crucial tool for identifying the active site. For Cu–ceria catalysts, the development of Cu–Ov–Ce_*x*_ structures under hydrogenation conditions is linked to methanol formation.^212^ The catalytic selectivity toward light olefins or hydrocarbons in Fe-based systems is determined by the Raman analysis of the gradual evolution from Fe_2_O_3_ to Fe_3_O_4_ and Fe_*x*_C_*y*_.^213^ Additionally, Raman measures Ov densities (via I_*D*_/IF_2*g*_), which, in CeO_2_-supported catalysts, scale with RWGS performance.^214^ Crucially, in situ Raman can distinguish subtle support-dependent reduction behaviors, as demonstrated in the Co_3_O_4_–ZnO system. Dong et al. showed that the characteristic Co oxide modes (E_*g*_, F_2*g*_, A_1*g*_) in mechanically mixed Co_3_O_4_–ZnO disappear at 400 °C under 10% H_2_/N_2_, whereas the same Co_3_O_4_ bands remain visible in pure Co_3_O_4_ even at 450 °C, indicating that the oxide persists under these conditions (Figure 9a).^215^ This evidence shows that ZnO accelerates the reduction of Co_3_O_4_, providing a mechanistic signature of the oxide–oxide interaction and highlighting Raman’s sensitivity to local interfacial confinement and spillover-induced reduction phenomena. Such Raman-derived fingerprints not only identify the active phase but also provide insights into the stabilization or destabilization of oxidation states, offering guidance that is relevant to catalyst design strategies across Ni-, Fe-, and emerging multicomponent systems.^211^

**Figure 9:**
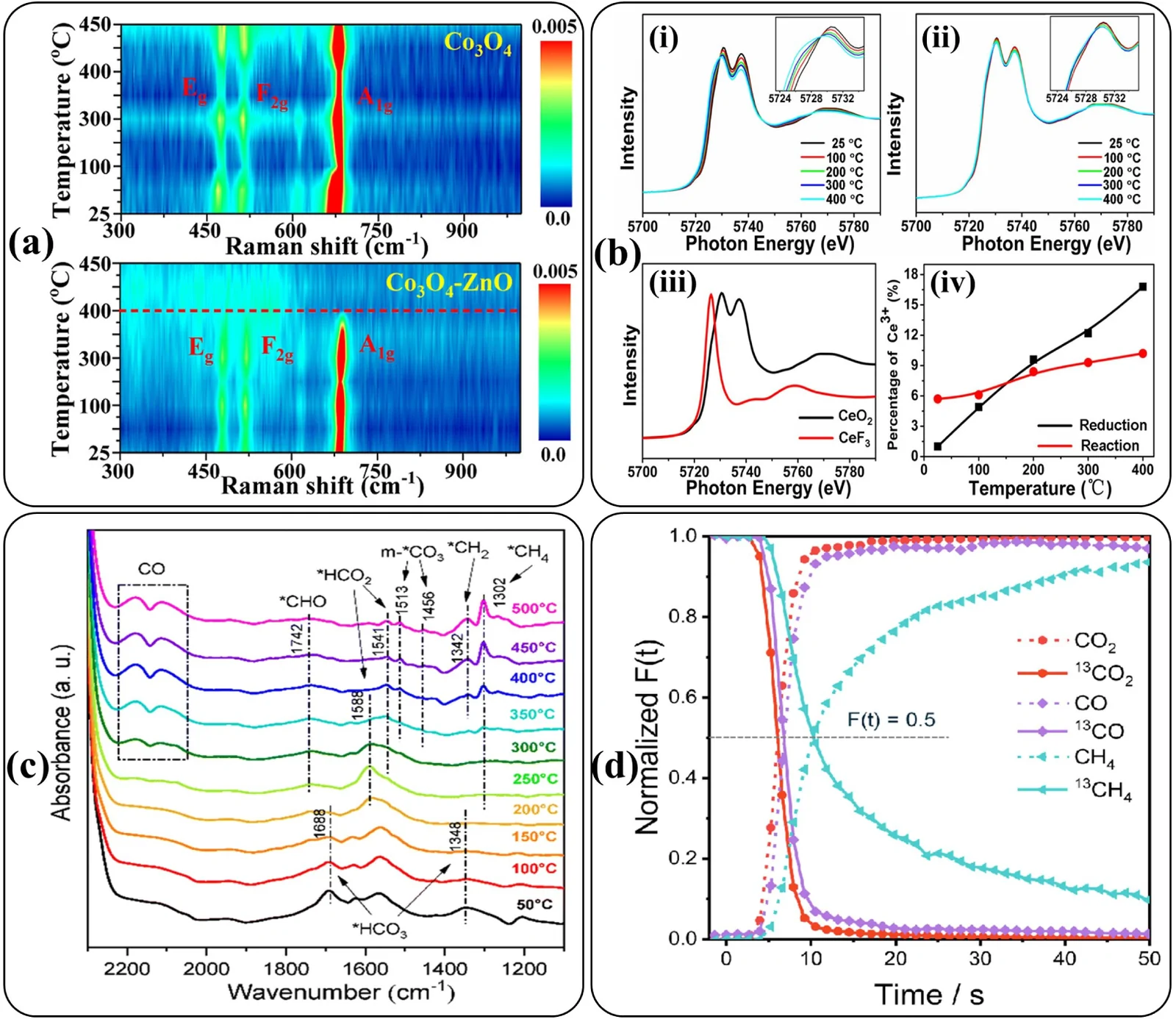
Operando and in situ methods reveal how catalytic surfaces reorganize under working conditions. (a) Raman spectra of Co_3_O_4_ and Co_3_O_4_–ZnO. Reproduced from ref 215. Copyright 2023, American Chemical Society. (b) XANES tracking of Ru/CeO_2_ captures the progressive reduction of ceria and the temperature-dependent shift in Ce^3+^ population, benchmarked against CeO_2_ and CeF_3_ standards. Reproduced from ref 216. Copyright 2016, American Chemical Society. (c) Temperature-resolved FTIR of Ni/SiO_2_ maps the appearance and evolution of key adsorbed intermediates as the surface is activated. Reproduced with permission from ref 222. Copyright 2023, RSC. (d) SSITKA switching between ^13^CO_2_ and ^12^CO_2_ after 4 h on stream exposes carbon turnover dynamics intrinsic to CO_2_–FTS pathways. Reproduced from ref 226. Copyright 2023, American Chemical Society.

Operando XANES/EXAFS provides complementary insight by quantifying redox changes and coordination evolution under working conditions. A representative example is the operando Ce L_3_-edge XANES investigation (Figure 9b, i–iv), where CeO_2_-based catalysts were monitored during both H_2_ pretreatment and CO_2_ hydrogenation.^216^ Ov-associated Ce^3+^ sites accumulate during reduction but partially reoxidize upon introduction of CO_2_, according to linear-combination fits using CeO_2_ and CeF_3_ references (Figure 9b, iii). This is consistent with the role of Ce^3+^ sites in adsorbing and activating CO_2_. This reversible redox cycle demonstrates that operando XANES can distinguish between reaction-induced reoxidation and reduction-driven vacancy formation, informing strategies to maintain optimal Ce oxidation levels. Across catalyst families, operando XAS consistently uncovers analogous working-state transformations. Multiedge XANES of Ni–Fe–Cu/SiO_2_ shows that Fe^0^ and Cu^+^ species selectively promote CO formation, whereas Ni^0^ governs overall CO_2_ conversion.^217^ In_2_O_3_/m-ZrO_2_ forms atomically dispersed In–O_V_–Zr motifs that suppress over-reduction and stabilize methanol-forming active sites.^218^ Comparable electronic restructuring has also been observed for Ru/CeO_2_ and other reducible-oxide systems. Collectively, these studies demonstrate that identifying—and stabilizing—the correct electronic state of the active site (Ni^0^, Fe^0^, Cu^+^, Ru^0^, and In^δ+^, etc.) is essential for directing selectivity and ensuring catalyst durability.

In situ/operando FTIR offers a direct window into the dynamic evolution of surface-bound intermediates, formate, monodentate/bidentate carbonates, carbonyls, methoxy, and CH_*x*_ species, that dictate whether CO_2_ hydrogenation follows RWGS, methanation, or methanol pathways.^219–221^ The technique excels in resolving intermediate lifetimes and interconversion sequences that govern selectivity. FTIR has elucidated how formate-derived CO can be split into CO or CH_4_ pathways by Ni-based catalysts, and how weak versus strong CO-binding sites influence methanation activity.^222^ Cu systems have dual *COOH and *CO pathways, where Cu^+^ stabilizes *CO and Cu^0^ activates H_2_. This electronic bifunctionality is detected by FTIR through different carbonyl signatures.^223^ In bifunctional GaZrO_*x*_/H-SAPO-17 systems, FTIR identifies methanol-derived polymethylbenzene hydrocarbon-pool species as the chain-growth centers for C_2_^+^ olefins,^224^ establishing a spectroscopic link between intermediate speciation and product slate. A representative FTIR example is provided by Figure 9c, where temperature-resolved FTIR (50–500 °C) on Ni/SiO_2_ shows the sequential disappearance of bicarbonate species, the emergence of monodentate carbonates and formate, and the appearance of *CHO, *CH_2_, *CH_4_, and linear/bridged *CO bands above ∼300 °C. This progression reveals that both RWGS and methanation pathways can coexist on larger Ni ensembles; however, methanation remains dominant due to enhanced hydrogenation activity. In contrast, Ni/SiO_2_ exhibits an RWGS-dominated profile (Figure 9c), where CH_X_ species are absent, and formate intermediates are more stable. Such operando FTIR evidence directly ties intermediate stability (e.g., persistent formate on confined Ni sites) to macroscopic CO selectivity.^222^ Across these systems, in situ FTIR provides important mechanistic insights for understanding catalytic behavior, including the identification of active intermediates, their temperature-dependent transformations, and the influence of particle size, oxidation state, and metal–support interactions on the balance among *COOH, *CO, and *CH_*x*_ pathways.

By combining FTIR with steady-state isotopic transient kinetic analysis (SSITKA), a quantitative kinetic technique, the rate-determining steps, surface residence periods, and coverages can be revealed.^211^ For Pd/Al_2_O_3_, SSITKA–FTIR revealed that *CO hydrogenation controls CH_4_ formation, whereas *COOH decomposition controls CO formation. The interaction between these processes manipulates product selectivity.^225^ SSITKA demonstrates significantly intricate mechanisms in Fe-based CO_2_–FTS catalysts. Normalized transient responses obtained after an isotope switch from ^13^CO_2_ to ^12^CO_2_ are shown in Figure 9d, which highlights different decay behaviors of CO and CH_4_. These profiles support previous interpretations of rising τCH_*x*_ and falling τCO with time on stream by confirming that CO and CH_4_ arise from parallel pathways occurring. Fe carbide formation is reflected in the SSITKA component analysis: the fast (short-lived) component decreases, while the slow (long-lived) component dominates, indicating the growth of a more persistent carbidic carbon pool during the reaction.^226^ Similarly, the different hydrogenation capacities of *CO on hexagonal-close-packed-structured Co versus face-centered-cubic-structured Co explain their divergent product distributions.^227^ These quantitative constraints provide a mechanistic basis that can guide catalyst design by linking performance to measurable parameters, rather than relying solely on empirical screening.

Across Raman, XAS, FTIR, and SSITKA, complementary insights are obtained that contribute to a more consistent mechanistic understanding of catalytic processes. The real active site is initially identified using in situ/operando tools, which then guide stabilization of its oxidation state or defect environment. These map the intermediates that should be inhibited or promoted and measure the kinetic bottlenecks that ultimately determine selectivity. Whether by improving oxygen vacancy density, modulating metal–support electron transport, or designing interfaces that favor specific intermediates, these mechanistic insights can guide catalyst development. As a result, operando spectroscopy can inform CO_2_ hydrogenation research by linking mechanistic understanding to structure-targeted catalyst design, thereby supporting the extension of design principles across catalyst systems.

### 4.2 Data-Driven Catalyst Design for CO_2_ Hydrogenation

#### 4.2.1 Foundations of ML-Assisted Catalyst Discovery

Artificial intelligence (AI) and machine learning (ML) are increasingly being explored in catalysis research as tools to accelerate catalyst discovery and improve the understanding of complex reaction networks.^228,229^ In systems such as CO_2_ hydrogenation, where multiple intermediates and competing pathways coexist, conventional density functional theory (DFT) approaches can become computationally demanding for large-scale screening. To address this, data-driven strategies have been developed that combine first-principles calculations with statistical and machine learning models to identify relationships between catalyst properties and performance.^228,230^ Data-driven catalyst design is primarily based on supervised machine learning, in which models are trained to establish quantitative relationships between catalyst descriptors (e.g., adsorption energies, electronic structure parameters) and performance metrics such as activity and selectivity.^228,231^ ML–DFT frameworks typically employ surrogate models to approximate adsorption energies and reaction energetics, thereby enabling more efficient exploration of reaction pathways and prioritization of kinetically relevant steps.^230^ A variety of model types, including regression-based approaches, kernel methods, and neural networks, have been reported in the literature for capturing structure–property relationships in catalytic systems.^228,231^ Model development typically involves descriptor selection, feature engineering, and validation strategies to ensure predictive accuracy and generalizability across catalyst systems.^229,231^ In parallel, descriptor-based screening and high-throughput computational approaches have been used to evaluate large catalyst spaces, with active learning strategies helping to iteratively improve model predictions and reduce computational cost.^228,229^ In contrast, unsupervised learning methods such as clustering are generally used for pattern recognition and feature extraction, playing a complementary but secondary role in catalyst design.^231^ These approaches represent the current state of the art in data-driven catalysis, enabling efficient screening, mechanism identification, and accelerated catalyst discovery.^228,229^ More recently, efforts have been made to integrate machine learning with high-throughput experimentation, enabling iterative workflows in which model predictions guide experimental design and data acquisition.^231^ These approaches are often implemented within closed-loop frameworks, although their level of automation and applicability can vary depending on the system under study. In this context, optimization strategies such as Bayesian optimization (BO) are frequently employed to guide exploration of the parameter space; however, their effectiveness is closely tied to the quality and representativeness of the underlying predictive models.^229^ Data-driven catalyst design approaches can generally be divided into two categories: (i) theoretical workflows, where models trained on computational data sets are used to predict catalyst structures and reaction energetics, and (ii) experimental workflows, where models are applied to guide synthesis conditions and catalytic testing.^231,232^ These approaches differ in terms of data requirements, scalability, and direct applicability to real-world catalyst development. Despite these advances, several challenges remain. These include the limited availability of high-quality and standardized data sets, the dependence of model accuracy on the underlying DFT calculations, and the difficulty of capturing dynamic catalyst behavior under reaction conditions.^228,229,231^ In the case of CO_2_ hydrogenation, these challenges are further compounded by the complexity of reaction networks, the coexistence of multiple competing pathways, and the potential for catalyst restructuring, all of which complicate the development of transferable descriptors and predictive models.^228,229^ Overall, AI/ML-assisted approaches provide promising tools for accelerating catalyst discovery and mechanistic understanding, although their successful application requires careful consideration of data quality, model limitations, and system-specific complexities.

In the context of CO_2_ hydrogenation, the application of data-driven inverse design presents additional challenges compared to more well-defined catalytic systems. This is primarily due to the complexity of reaction networks and the coexistence of multiple competing pathways (e.g., RWGS, methanation, methanol synthesis, and C_2_^+^ formation), as widely discussed in mechanistic studies of CO_2_ conversion.^19,130,233^ These factors, together with catalyst restructuring under reaction conditions, limit the transferability of descriptors and reduce the predictive accuracy of machine learning models, particularly when trained on static data sets.^230,234^ As a result, catalyst design in CO_2_ hydrogenation remains more system-specific compared to simpler catalytic reactions.^228^ Future progress is expected to rely on the integration of operando data, improved descriptor development, and hybrid frameworks combining first-principles calculations with machine learning to better capture reaction dynamics.^231,235^

Overall, these advancements lay the groundwork for data-driven catalyst design, which combines high-throughput procedures, machine learning models, and catalytic descriptors to speed up catalyst discovery. These ideas have led to the development of inverse-design techniques that actively direct the investigation of catalyst composition and synthesis space. Because BO can effectively identify high-performing catalysts while requiring the least experimental work, it has attracted the most interest among these methods.

#### 4.2.2 Bayesian Optimization and Closed-Loop Catalyst Discovery

Due to its widespread use in closed-loop catalyst development processes, BO is presented here as a representative example of the various inverse-design methodologies documented for catalyst discovery. However, as other techniques, including genetic algorithms, evolutionary optimization, active-learning frameworks, and random-search strategies, have also been successfully used in catalyst design, BO should not be seen as the only recommended way. In this broader framework, new research shows how BO and machine learning can transform the development of CO_2_-hydrogenation catalysts from empirical trial and error to data-driven optimization.

A core advance behind this shift is the development of descriptors that capture catalytic complexity that extends well beyond single-site physics. Adsorption-energy distributions (AEDs), a statistical fingerprint created from thousands of adsorption energies measured across surfaces, facets, and important intermediates (*H, *OH, *OCHO, and *OCH_3_) pertinent to CO_2_-to-methanol conversion, were presented by Pisal et al.^236^ More than 877,000 adsorption energies for 158 alloys were calculated in this study using machine-learned force fields. Unsupervised clustering was used to identify compositional families whose AED profiles resemble those of well-known high-performing catalysts, revealing previously unidentified candidates such as ZnRh and ZnPt_3_. To frame this approach, the workflow illustrated in Figure 10a highlights the systematic pathway from catalyst structure to adsorption-energy landscapes. AEDs ultimately demonstrate how ML can characterize entire catalytic landscapes rather than isolated adsorption sites, providing a foundation for more holistic and scalable catalyst-design strategies. Closed-loop BO is used to accelerate experimental discovery. While BO is widely used for efficient exploration of catalyst design space, the predictive capability of the underlying machine learning model remains the primary determinant of performance.^229–231^

**Figure 10:**
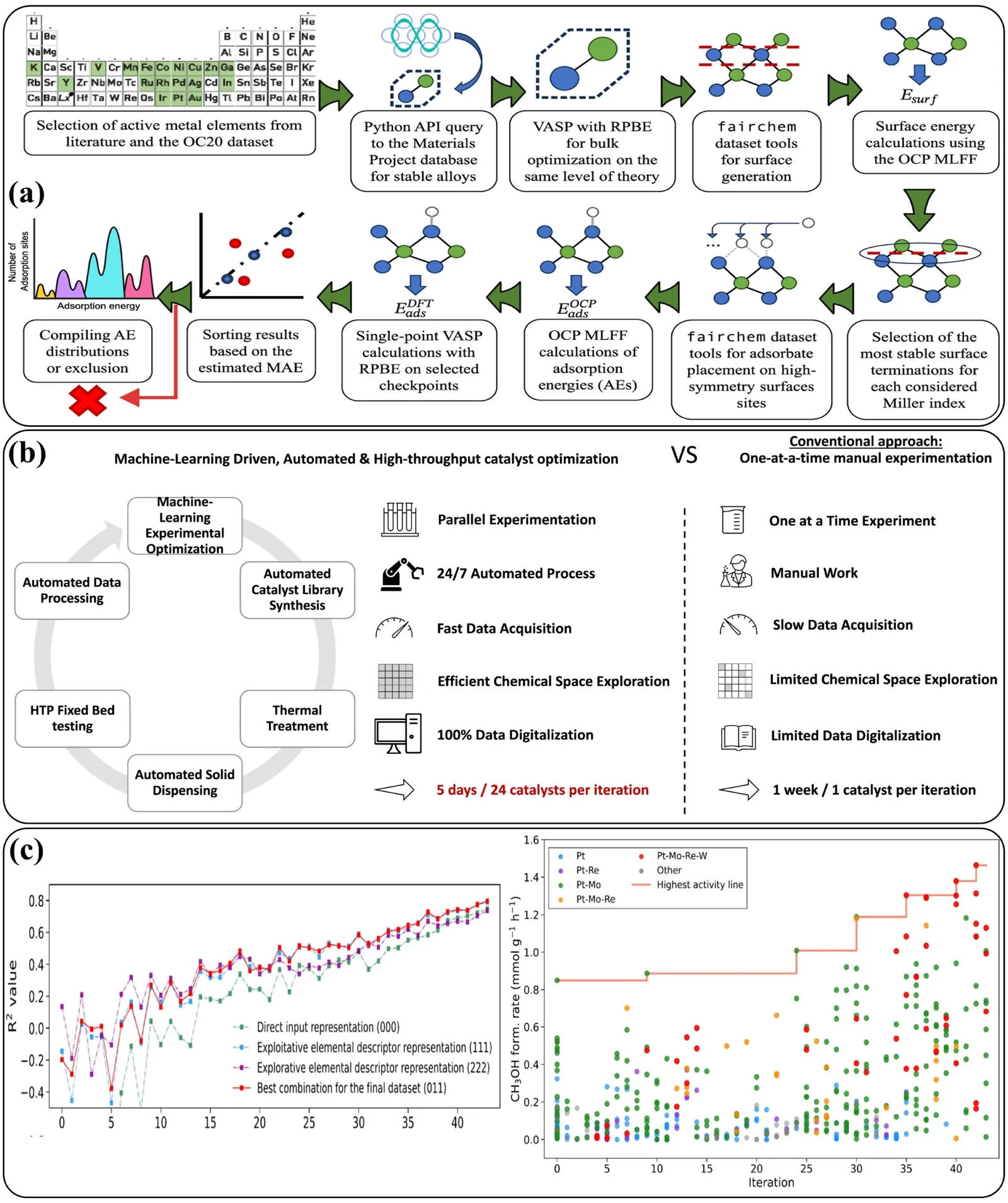
(a) Workflow for generating adsorption-energy distributions (AEDs). Reproduced from ref 236. Available under a CC-BY 4.0 license. Copyright 2025, Springer Nature. (b) Overview of the closed-loop BO framework, which highlights the integration of automated synthesis, high-throughput testing, and ML-driven decision-making compared with conventional iterative approaches. Reproduced with permission from ref 237. Copyright 2024, Elsevier. (c) Iterative ML-guided catalyst investigation demonstrates that prediction accuracy improves with data set size and evolving descriptor representations. The maximum methanol-synthesis activity is found in each iteration, and catalysts that incorporate novel elemental combinations are distinguished from those in the original design space using symbols. Taken as a whole, the progression shows that increasingly accurate navigation of complex multimetallic catalyst space is enabled by adaptive representation and growing experimental feedback. Reproduced from ref 241. Copyright 2025, American Chemical Society.

If descriptor engineering provides the map, BO supplies the engine for navigating it. Ramirez et al.^237^ combined BO with thermal treatment, automated solid dispensing, robotic synthesis, and high-throughput fixed-bed testing to perform fully autonomous catalyst optimization for CO_2_-to-methanol hydrogenation. In a closed-loop discovery, the system iteratively proposed, synthesized, and evaluated 144 catalysts across six generations in 6 weeks, progressively converging on high-performing Cu–Zn–Ce/ZrO_2_ formulations. This ML-guided cycle delivered a 5.7-fold increase in CO_2_ conversion, a 12.6-fold enhancement in methanol formation, and a 3.2-fold reduction in CH_4_ selectivity, while simultaneously achieving a 6.3-fold decrease in metal cost. Achieving a multiobjective improvement was nearly impossible with conventional trial-and-error optimization. Figure 10b illustrates a machine learning–driven, automated catalyst discovery workflow in which synthesis, testing, and data processing are integrated into an iterative loop, with BO guiding the selection of subsequent experiments. These results show that BO can reduce years of trial-and-error screening to a few weeks by efficiently exploring many compositions while focusing attention on the most promising ones as they emerge. BO provides an efficient framework for iterative selection of experiments within model-guided workflows.^238^

Recent research demonstrates that BO can effectively guide catalyst synthesis. Wen et al.^239^ applied BO to optimize the calcination and reduction conditions for silica-supported Ni catalysts derived from nickel phyllosilicates. It identified synthesis parameters that maximize the balance between metallic Ni^0^ and Lewis-acidic Ni^x+^ sites. Spectroscopic investigation revealed a strong, unexpected correlation between the Ni coordination environment and the synthesis conditions, and the improved catalyst exhibited significantly higher activity. In addition to ML techniques for catalyst design, this study highlights that high-order synthetic dependencies are captured by machine learning models, with BO facilitating their efficient exploration. BO also has demonstrated transformative capabilities through gradual, empirical tuning. Applying multivariate BO to CoO nanoparticle synthesis, Karadaghi et al.^240^ identified complex, high-order correlations between synthesis parameters, with these relationships captured by the machine learning model and navigated through the optimization process. When supported on SiO_2_, the improved synthesis yielded monodisperse, phase-pure CoO nanocubes with ∼98% CH_4_ selectivity and strong high-temperature stability, demonstrating the apparent correlation between catalytic performance and nanoparticle design. By using extrapolative models based on weighted elemental descriptors, Zhao et al.^241^ extended ML-driven catalyst discovery. They enabled accurate forecasting of catalyst performance even for compositions that lacked elements in the original training data set. Approximately 580 multimetallic catalysts were investigated across 43 iterative ML-experiment cycles before identifying Pt–Mo–Re–W/TiO_2_ as a highly active low-temperature methanol-synthesis catalyst. The development of prediction accuracy across iterations is shown in Figure 10c, which emphasizes how model performance was impacted by various descriptor representations (000, 111, 222, and 011) as the data set expanded. The prediction accuracy (R^2^, MAE, RMSE, MSE) remained low in the early iterations, when the training data were limited and chemically varied, and only the 222 representation was employed. Despite the challenging initial data landscape, the model gradually improved predicted reliability and enabled efficient catalyst identification by using 111, 222, and the best-performing representation at each iteration. A cooperative ensemble mechanism, such as Pt driving H_2_ activation, Mo producing oxygenated intermediates, Re accelerating formate hydrogenation, and W promoting methanol desorption, was discovered through operando analysis of the final ML-selected catalyst. It highlights how mechanistic insight can validate and improve ML-derived predictions.

By using sophisticated descriptors to capture catalytic complexity across sites, facets, and intermediates, these findings establish a new integrated paradigm for catalyst discovery. Multivariable exploration is then accelerated from years to weeks using closed-loop BO and ML-guided screening. In addition to BO, other optimization strategies, such as genetic algorithms, evolutionary methods, and random search, have also been applied to catalyst design.^231,232,242^ AI-driven methods reveal nonintuitive compositions and elucidate higher-order relationships in nanoscale synthesis by enabling rapid exploration of high-dimensional design spaces. This enables simultaneous optimization of activity, selectivity, and cost by expanding the accessible chemical space well beyond human intuition. However, these developments also reveal critical limitations. Model dependability remains severely limited by descriptor selection, data quality, and underlying physical assumptions. Further, early stage models often exhibit poor predictive accuracy when trained on sparse or chemically diverse data sets. Therefore, iterative data enrichment, uncertainty-aware model selection, and strong integration with operando and mechanistic validation are critical to the efficacy of AI-driven acceleration. This algorithmic technique transforms CO_2_-hydrogenation catalyst discovery from incremental empirical tuning to rapid, comprehensible, and mechanistically grounded design when combined with high-quality experiments and qualified human oversight.^236,237,240,241^

### 4.3 Section Summary and Design Implications

Machine learning provides a powerful framework for translating mechanistic descriptors into predictive models and accelerated catalyst discovery. By encoding features such as adsorption energies, intermediate stability, and surface composition, ML models can map complex catalytic landscapes beyond what intuitive design can capture. Closed-loop optimization strategies further enable iterative refinement of catalyst composition and operating conditions. However, the effectiveness of these approaches critically depends on data quality, descriptor selection, and integration with mechanistic understanding. In this context, AI/ML does not replace fundamental catalysis but operationalizes it, transforming descriptor-based insights into actionable design strategies. Importantly, the targets defined by these models must be aligned with process-level constraints to ensure that optimized catalysts remain relevant for real-world deployment.

## 5 Catalytic Reactor Design and Process Intensification

### 5.1 Structured and Electrified Reactors for Selectivity Control

Reactor design in CO_2_ hydrogenation is fundamentally governed by transport and energy-transfer phenomena, including heat and mass transfer, residence time distribution, temperature gradients, and hydrodynamic effects, which can obscure intrinsic catalytic behavior and influence reaction pathways, thereby affecting product selectivity.^243–247^

Reactor configurations such as structured reactors are therefore selected to control these phenomena by minimizing transport limitations and enabling near-isothermal operation.^248,249^ By reducing characteristic transit lengths and enhancing heat and mass transfer, structured reactors, such as microstructured packed beds and monolithic architectures, mitigate these limitations.^247,250–252^ Rapid heat removal inhibits hot-spot formation and catalyst sintering, which are frequently observed in traditional packed beds, particularly in CO_2_ methanation and methanol synthesis.^246^ In circumstances where conventional systems favor methane, structured reactors provide more reliable access to oxygenate-forming pathways by maintaining near-isothermal operation in kinetically regimes.^247,252^ The microstructured packed-bed reactor (Figure 11a), in which enhanced heat and mass transfers enable precise kinetic measurements without interference from external transport constraints, illustrates how reactor structuring contributes to kinetically controlled selectivity.^251^ These reactor designs provide realistic platforms for examining structure–selectivity interactions in CO_2_ hydrogenation by separating inherent catalytic selectivity from reactor-scale influences.^247,250–252^

**Figure 11:**
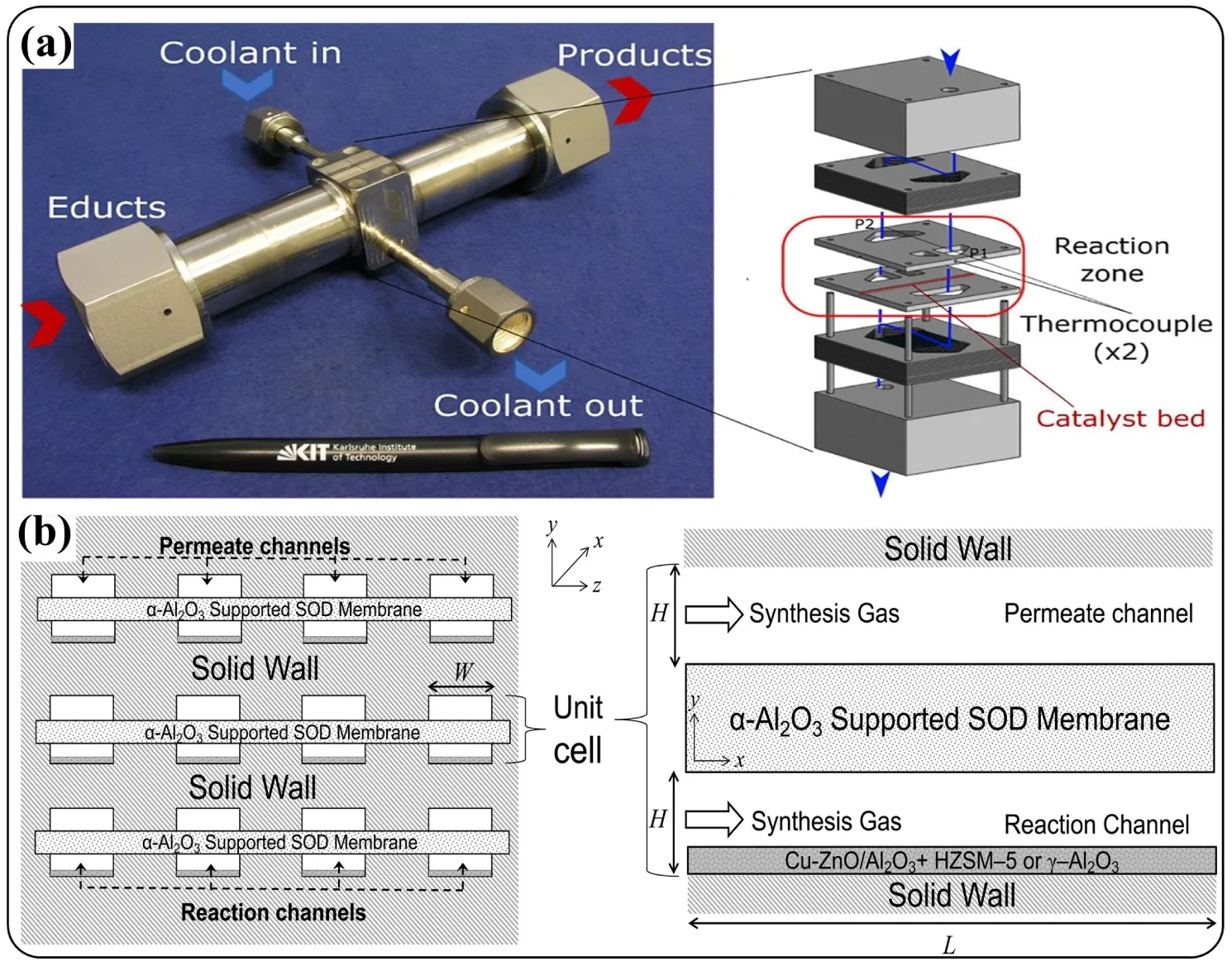
(a) Schematic of a microstructure packed-bed reactor featuring intensified heat and mass transfer, employed for kinetically controlled CO_2_ hydrogenation studies. Reproduced with permission from ref 251. Copyright 2020, Elsevier. (b) Conceptual illustration of a membrane microreactor for CO_2_ hydrogenation, showing the integrated reaction–separation architecture (left) and the corresponding unit-cell configuration highlighting the catalyst-coated channels and selective membrane layer (right). Reproduced with permission from ref 255. Copyright 2022, Elsevier.

Control of thermodynamic limitations can be achieved through reaction–separation coupling, as demonstrated in membrane microreactors.^253–255^ These systems incorporate selective membranes that continually extract products into catalyst-coated reaction channels. Under mild operating conditions, the membrane microreactor (Figure 11b) demonstrates that in situ water removal alters thermodynamic equilibria, reduces RWGS, and increases methanol or DME yields.^255,256^ Membrane microreactors efficiently decouple conversion from selectivity by spatially coupling reaction and separation, thereby overcoming equilibrium limitations.^253,254^

Beyond conventional thermal heating, the key distinction lies in how energy is transferred into the reaction medium, including thermal, microwave, and plasma-assisted modes. These energy-transfer modes provide additional degrees of freedom for tuning reaction pathways and selectivity beyond conventional operation.^257–259^ Rapid, volumetric, and catalyst-selective heating is enabled by microwave reactors, which reduce temperature gradients between the solid and gas phases.^246,260^ Under nonequilibrium conditions, electrically or vibrationally excited CO_2_ species encourage alternative activation pathways that favor CO production or inhibit deep hydrogenation in microplasma reactors.^261,262^ These systems demonstrate that electrical characteristics, such as power input, frequency, and discharge configuration, can be used to adjust selectivity, rather than relying on catalyst modifications.^261–263^

Despite their potential, large-scale deployment of electrified reactors remains constrained by plasma stability, energy efficiency, and reactor durability, underscoring the necessity of integrated reactor–catalyst optimization.^246^ Overall, reactor design controls transport and energy-transfer phenomena, transforming the reactor from a passive vessel into an active element that influences reaction pathways and product selectivity.^245,246,260^ These devices are well-positioned as ideal platforms for integrating data-driven optimization strategies in emerging CO_2_ hydrogenation systems, owing to their compatibility with operando diagnostics, enhanced transport capabilities, and automated control.^264–266^

### 5.2 Integration with Renewable Hydrogen and Circular Carbon Systems

Integrating CO_2_ hydrogenation with renewable hydrogen production and circular carbon systems has become crucial for translating catalytic advances into greenhouse gas reduction. Recent studies emphasize that CO_2_ hydrogenation must be considered within an integrated energy–carbon system. In this framework, hydrogen produced from renewable electricity is mixed with captured CO_2_ to produce fuels or chemicals, which are then re-emitted and recaptured after final usage.^267,268^ CO_2_ reduction through renewable hydrogen is a key to mitigating anthropogenic carbon emissions (Figure 12).

**Figure 12:**
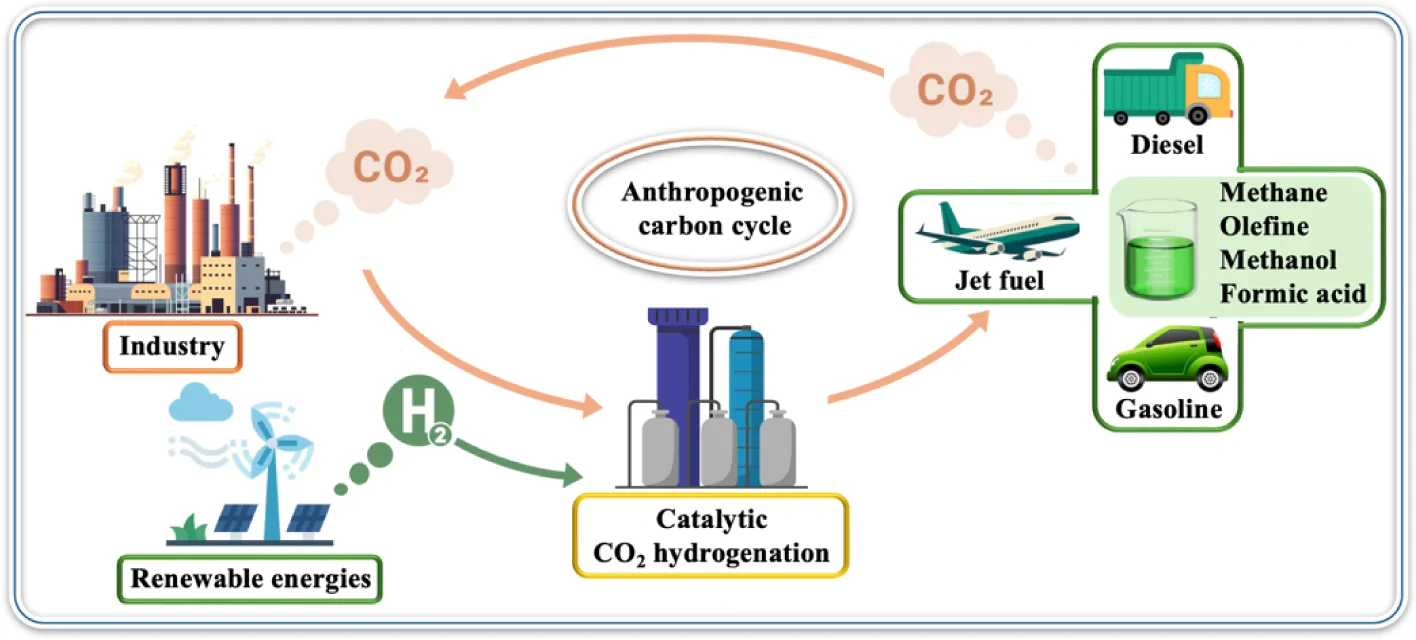
Integration of catalytic CO_2_ hydrogenation with renewable hydrogen production to close the anthropogenic carbon cycle through fuels and chemical synthesis.

The carbon footprint and cost structure of CO_2_ hydrogenation methods are primarily determined by the hydrogen supply, according to techno-economic and life-cycle assessments. When fossil fuels are used to produce hydrogen, upstream emissions may exceed the benefits of CO_2_ reductions, undermining the process’s carbon-neutrality.^269^ Thus, CO_2_ hydrogenation is repositioned as a Power-to-X technology that links intermittent renewable power to chemical energy carriers, and hydrogen produced by water electrolysis using low-carbon or renewable electricity is considered essential for sustainability.^267–269^ Modern electrolyzers’ operational flexibility enables the integration of downstream hydrogenation reactors and variable renewable electricity supplies.

Methanol synthesis is commonly described as a promising option for circular carbon integration among CO_2_ hydrogenation pathways because of its dual function as a chemical feedstock and a liquid energy carrier. Methanol produced from collected CO_2_ and renewable hydrogen can be readily integrated into existing chemical infrastructure, enabling large-scale storage and transportation of renewable energy.^269^ However, single-pass CO_2_ conversion is limited by equilibrium constraints, necessitating elaborate recycle loops, purge control, and heat integration. System simulations highlight the interdependence of catalysis and process integration by demonstrating that high overall CO_2_ utilization and favorable carbon efficiency can be achieved only when catalyst performance is adjusted in tandem with reactor design, separation procedures, and recycle architecture.^267–269^ The system-dependent character of CO_2_ hydrogenation is further highlighted by demonstration-scale implementations. A typical example of how abundant low-carbon electricity allows the conversion of captured CO_2_ into methanol with significantly lower life-cycle emissions is the geothermal methanol plant run by Carbon Recycling International in Iceland.^267^ However, because the climatic benefits and scalability of CO_2_ hydrogenation are closely linked to local renewable energy supply and CO_2_ sources, these instances highlight geographical limitations.^268^

The net environmental impact of CO_2_ hydrogenation is sensitive to the carbon intensity of the electricity used to generate hydrogen, as life-cycle assessment and techno-economic studies have consistently shown. Depending on whether electricity is supplied by fossil-fuel-dominated, low-carbon, or renewable grids, identical catalytic devices can switch from net CO_2_ mitigation to net emissions.^270,271^ These results encourage a shift away from reaction-centric performance metrics toward integrated system-level evaluation criteria that account for grid emissions, CO_2_ capture, catalyst activity, electrolyzer efficiency, and heat and mass integration across the entire process chain.^270^ According to the study, CO_2_ hydrogenation can yield sustainable outcomes only when integrated into circular carbon systems and renewable hydrogen supply chains, where catalytic innovation and energy-system design are developed concurrently.^268^

## 6 TEA and LCA Benchmarks for CO, CH_4_, And C_2_^+^ Hydrocarbons

Life-cycle assessment (LCA) and techno-economic analysis (TEA) are the primary interpretation tools for CO_2_ hydrogenation. Advances in activity, selectivity, and stability at the catalyst level are translated into system parameters, including life-cycle carbon intensity, energy intensity, and minimum selling price (or levelized cost). Clear system boundaries (gate-to-gate to cradle-to-grave) and specific assumptions for the supply of power, H_2_, and CO_2_ are required when reporting these results.^272,273^ System-level economics and climate performance are rarely limited by the hydrogenation reactor alone, according to existing TEA/LCA benchmarking studies. The possible cost-carbon envelope for CO_2_-derived products is instead largely determined by upstream and plant-wide levers, such as (i) the cost and carbon intensity of electricity and electrolytic H_2_, (ii) CO_2_ sourcing and purification along with the selected crediting convention, (iii) the degree of process integration that valorizes heat and carbon, such as steam export, tail-gas recycle, and (iv) scale, financing assumptions, and capacity utilization.^273–275^ The TEA/LCA-defined operating window for CO_2_ hydrogenation is summarized in Figure 13, where CO, CH_4_, and C_2_^+^ hydrocarbons are treated as benchmark targets whose availability is determined by system-level constraints rather than by performance alone.

**Figure 13:**
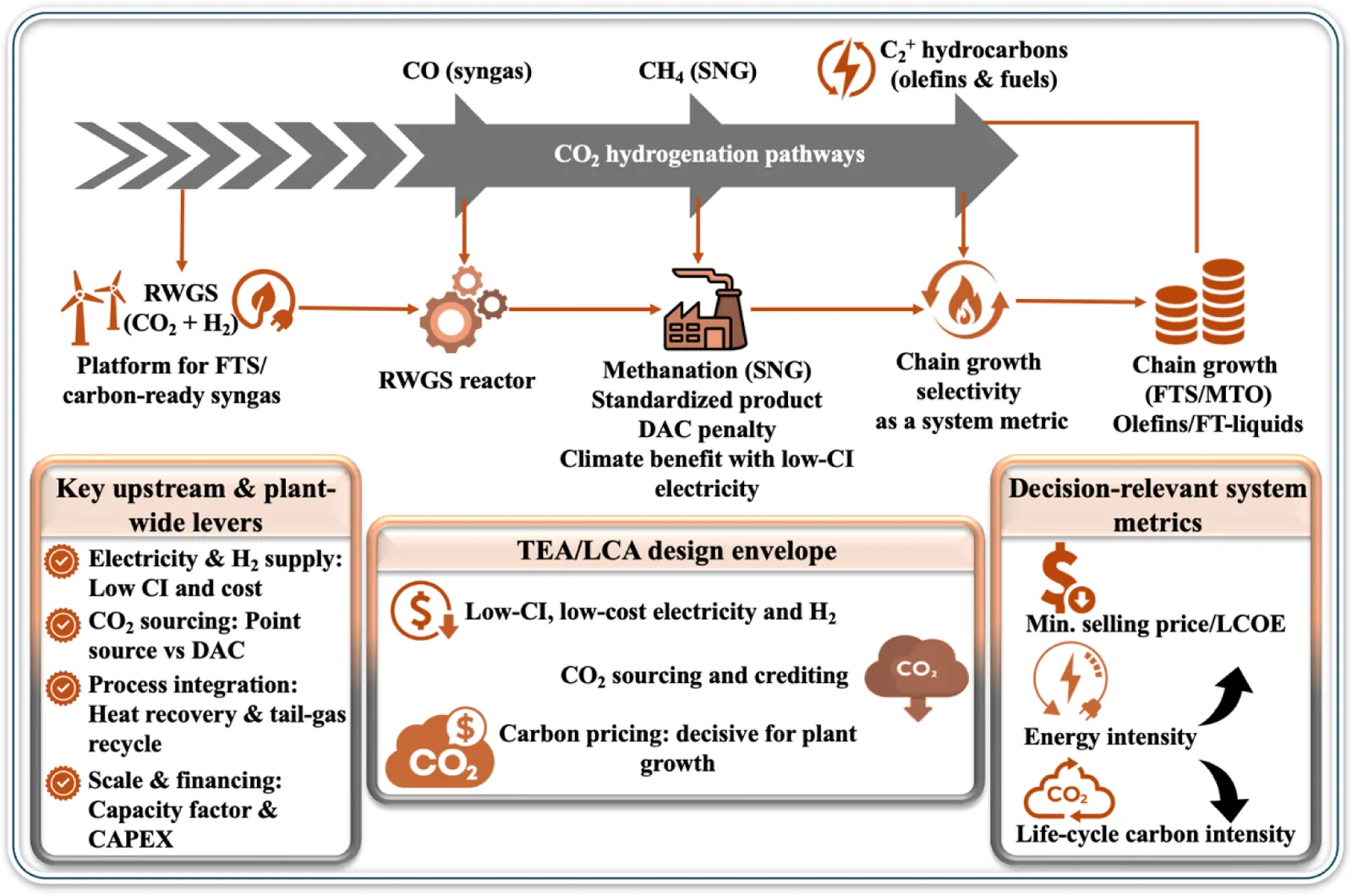
TEA/LCA-defined operating window for CO_2_ hydrogenation to CO, CH_4_, and C_2_^+^ hydrocarbons.

In addition to these considerations, product selectivity plays a central role in determining process-level performance in CO_2_ hydrogenation.^37,276^ Methane formation, while thermodynamically favorable, offers limited carbon utilization efficiency and depends strongly on low-carbon hydrogen availability.^276,277^ In contrast, methanol synthesis benefits from a relatively narrow product distribution, resulting in lower separation energy and improved process integration.^278,279^ C_2_^+^ hydrocarbons, although higher in value, typically exhibit broad product distributions governed by Anderson–Schulz–Flory behavior, leading to increased separation and recycle requirements, which elevate energy demand and process complexity.^37,280^ These differences highlight that techno-economic and life-cycle performance are strongly governed by the interplay among selectivity, separation energy, and carbon intensity.^37,276,279^ The cost and carbon intensity of electricity-based H_2_, as well as the sourcing and crediting of CO_2_, largely determine the primary outcomes in TEA and LCA across products. The overall carbon cost window is further shaped by plant scale and financing, as well as by process integration, such as recycling, heat recovery, steam generation, and utility use. Benchmarks for CO (syngas) as a feedstock for FTS or methanol-to-olefins (MTO) are frequently published (e.g., RWGS to CO-rich syngas), as high H_2_ prices increase the cost of downstream products. The cost of syngas production can vary substantially within a direct air capture (DAC)-to-olefins framework, depending on the syngas generation route and underlying assumptions, such as whether production is electrochemical or thermochemical. The minimum cost level for fuels and CO-based compounds is therefore determined by the cost of syngas.^281^ The key characteristics are observed in other system-level TEA investigations of CO production. The competitiveness of CO products can be significantly affected by lower capital expenditure (CAPEX) through learning, improved catalyst or absorber performance, and the projected carbon price or penalty.^282,283^ The CO benchmarks are more useful as a carbon-ready syngas price rather than as a fixed $/kg of CO value. Depending on the system boundary, such as the source of CO_2_, the carbon intensity of energy, and the treatment of coproducts, the results can vary significantly.^272,273^

Because CH_4_ is a standardized commodity with extensive LCA data available, TEA and LCA benchmarks for CO_2_ methanation can be more straightforwardly compared by referencing CH_4_, i.e., synthetic natural gas (SNG). According to a power-to-methane study that combines methanation and solid-oxide electrolysis (SOE), syngas production accounted for 81.3–83.4% of the CH4 unit cost. The unit costs for onshore wind, offshore wind, and solar PV decreased from 0.106/0.125/0.114 $·MJ^–1^ to 0.082/0.100/0.089 $·MJ^–1^ as plant size increased from 1 MW to 10 MW. For the same power cases, the same study reported cradle-to-gate net climate impacts of −0.030/–0.030/–0.015 kg CO_2_-eq·MJ^–1^ at 1 MW and −0.031/–0.030/–0.016 kg CO_2_-eq·MJ^–1^ at 10 MW.^275^ A second TEA demonstrated a clear cost penalty for employing CO_2_ obtained via DAC and linked DAC to methanation. When CO_2_ was produced by a CaL-based DAC unit, the levelized cost of methane (LCOM) is approximately 4.9–8.2 €·kgCH_4_^–1^ at an electricity price of 0.1 €·kWh^–1^. Instead, LCOM is reduced to ∼3.1–3.9 €·kgCH_4_^–1^ with point-source capture (PSC). The levelized cost of CO_2_ removal by DAC (LCCR_DAC_) in the same study ranged from around 693 to 1587 €·tCO_2_^–1^ across best- and worst-case scenarios. This demonstrated that the cost of CO_2_ production can have a greater influence on overall economics than the methanation reactor step.^274^

The multistudy synthesis in Garcia–Garcia et al.^273^ established a crucial benchmark criterion on the LCA side: methane from CO_2_ is climate-beneficial only when energy is sufficiently low-carbon. CO_2_-based methane can achieve a cradle-to-grave carbon footprint of ∼14.1 g CO_2_-eq·MJ−1 ^284^ and consumes ∼15.2 MWh per ton of CH_4_.^244^ Depending on the system boundary and crediting assumptions, using wind electricity can meet carbon-neutrality targets. There was also an energy carbon-intensity (CI) threshold of around 113 g CO_2_·kWh^–1^, above which power-to-gas methane loses its climate benefit.^273^ According to an earlier power-to-gas LCA, replacing fossil natural gas with renewable power-to-methane can significantly reduce greenhouse gas impacts (∼44% reduction), but overall benefits remained limited by methanation efficiency losses and CO_2_ separation burdens.^285^

Selectivity is important not only for the catalyst but also for the overall process cost and life-cycle emissions of C_2_^+^ hydrocarbons (olefins and FT-range liquids) produced from CO_2_. CO_2_ capture, H_2_ production, conversion to CO (RWGS) or direct hydrogenation, and chain growth (FTS) or olefin synthesis (MTO-type methods) are required for these pathways. Due to this complexity, heat recovery and recycling streams (tail-gas recycling) frequently exert a significant influence on TEA and LCA outcomes.^272,281,286^ In the optimal scenario (90% tail-gas recycle), a CO_2_-to-jet-fuel process study (CO_2_–FTS with tail-gas recycle) estimates a minimum selling price (MSP) of ∼US$2.6 kg^–1^ jet fuel and an energy demand of ∼4.57 MWh t^–1^, resulting in ∼85.8% CO_2_ conversion and ∼35.6% jet fuel yield.^286^ Zang et al.^287^ estimated an MSP of $5.4–5.9 per gallon for FT fuel and compared their findings with previous CO_2_–FTS TEA research. Other studies, such as the 73–128 M$ CAPEX and 244–1951 M$/yr operating expenditure (OPEX) published by Fernández-Torres et al.^288^ for CO_2_–FTS gasoline systems, demonstrated significant cost uncertainty at commercial scale. According to a DAC-to-olefins analysis, olefin production is challenging because single-pass selectivity is not the only target; energy and carbon efficiency must also be optimized. The study presented energy-intensity and carbon-efficiency values that demonstrated considerable dependence of olefin performance on the overall process design. Therefore, in both TEA and LCA, olefins remained among the more challenging C_2_^+^ targets.^281^ According to certain electrofuel LCA, C_2_^+^ benchmarks should be compared with fossil-fuel or regulatory baselines. A life-cycle carbon intensity of ∼92 g CO_2_-eq·MJ^–1^ was reported in an FT electrofuel study employing H_2_ and ethanol byproduct CO_2_.^289^ The reference fuel, the allocation/system-expansion technique, and the carbon intensity of the power all affect the estimated life-cycle carbon intensity/greenhouse-gas footprint of the CO_2_-hydrogenation product.^272,273,290^

These TEA/LCA benchmarks provide a distinct design window for CO_2_ hydrogenation to CO, CH_4_, and C_2_^+^ products. First, the primary drivers of cost and emissions are H_2_, low-cost electricity, and low-carbon electricity. Second, the credit counting method and the CO_2_ source (point source vs DAC) may affect the outcome. Third, advances in catalysts are translated into tangible plant-level benefits through process integration, such as heat recovery, steam export, and tail-gas recycling. Lastly, for low-carbon scenarios to be economically competitive, carbon price or policy credits might be required.^273–275,286^ Single-pass conversion and selectivity, acceptable H_2_ use, and recycling ratio, should be determined using TEA and LCA as design constraints. This is crucial for C_2_^+^ products, as multistep integration increases the process’s sensitivity to upstream energy and efficiency losses.^272,290,291^

Techno-economic and life-cycle assessments provide the boundary conditions that determine whether catalytic advances translate into practical technologies. Across CO, CH_4_, methanol, and C_2_^+^ pathways, selectivity directly governs hydrogen consumption, separation energy, process complexity, and carbon intensity. While certain reaction pathways may be kinetically favorable, their large-scale viability is constrained by downstream processing penalties and energy demand. Therefore, catalyst design must be guided not only by mechanistic understanding and data-driven optimization but also by these system-level constraints, which define realistic deployment targets. These process-level constraints directly connect mechanistic descriptors and AI-driven catalyst design to real-world feasibility.

## 7 Outlook and Roadmap

This review integrates catalyst chemistry, reaction pathways, operando characterization, and emerging AI and ML tools to frame CO_2_ hydrogenation as a system-level design problem. TEA and LCA are used to evaluate whether laboratory-scale selectivity gains remain meaningful once hydrogen demand, electricity-carbon intensity, separation and recycle requirements, thermal management, and catalyst stability are considered. The central message is clear: Selectivity has value only when scale-up penalties are taken into account. CO_2_ hydrogenation is not a single reaction but an interconnected network in which RWGS, methanation, and C_2_^+^ formation compete for shared carbon and hydrogen pools. Small changes in surface-state or local H_2_-to-CO_2_ ratios can therefore shift the dominant pathway. As a result, selectivity under disturbance, rather than steady state performance alone, emerges as a key design criterion for translating laboratory advances into deployable technologies. In this context, selectivity under disturbance refers to a catalyst’s ability to maintain a stable product distribution under dynamic or nonideal operating conditions, such as temperature, feed composition, or transient operation.

Across Ni-, Fe-, and Cu-based catalyst systems, as well as emerging catalyst systems, a shared design logic is emerging. Catalyst development must be guided by dominant failure modes rather than peak activity. Here, dominant failure modes denote the primary processes that limit catalyst performance, including sintering, coking, phase transformations, oxidation-state drift, and selectivity loss under reaction conditions. For Cu-based catalysts, maintaining Cu^0^/Cu^+^ balance, preventing sintering, and controlling metal–support interactions are critical for sustaining methanol selectivity under realistic operating conditions. For Ni catalysts, controlling hydrogenation intensity and heat release is often as important as improving intrinsic kinetics. For Fe-based and tandem pathways, sustained selectivity depends on phase stability, water tolerance, and carbon management, not solely on enabling C–C coupling. New materials, including carbides, phosphides, and entropy-stabilized oxides, expand the design space for redox and defect control but must be evaluated by their ability to maintain performance over time. Building on these advances, future catalyst development is expected to increasingly leverage emerging material platforms and integrated design strategies. High-entropy materials provide tunable multielement compositions that enable precise control over adsorption properties and stability, while transition metal phosphides offer unique electronic structures that can balance hydrogenation activity and selectivity. In parallel, computational–experimental closed-loop approaches that combine density functional theory, machine learning, and high-throughput experimentation are emerging as powerful tools for accelerating catalyst discovery and optimization. These strategies enable iterative refinement of catalyst design by linking predictive models with real-time experimental validation. Operando and time-resolved measurements, therefore, become essential system diagnostics, enabling early detection of deactivation during redox cycling, steam exposure, and transient operation. Data-driven approaches are effective only when such stability and selectivity descriptors are incorporated into model training. At the process level, transport and thermal management strongly shape selectivity outcomes. Structured reactors, monoliths, and electrified or actively heated designs are increasingly important for mitigating gradients, particularly in exothermic methanation and C_2_^+^ synthesis, where broad product distributions can drive energy demand through separation and recycling. Co-design of catalyst, reactor, and separation is thus central to defining deployable operating windows.

The link between promising chemistry and reliable implementation lies in the integration of renewable H_2_ and circular carbon systems. When CO_2_ hydrogenation is combined with scalable electrolysis and low-carbon electricity, transparent CO_2_ sourcing (point source or DAC), and practical infrastructure for product transport and CO_2_ recapture, it becomes the most viable option. Crucially, intermittency is significant because load-following operation modifies catalyst states, coverages, and temperatures. Therefore, ramping and start–stop techniques that replicate renewable-powered operation should be used to verify durability and selectivity.

The governing restrictions that link all these threads are provided by TEA and LCA. The TEA and LCA results from previous studies consistently identify the same key elements. The baseline cost and carbon intensity are determined by the availability of electricity and H_2_, but the end competitiveness can be significantly altered by CO_2_ sourcing/crediting, process integration, scale, and financing. The cost of carbon-ready syngas is the most useful metric for assessing CO benchmarks. CH_4_ benchmarks are more comparable but highly electricity-dependent. C_2_^+^ benchmarks necessitate careful consideration of separation and recycle penalties. In the future, the most convincing research will reveal not only catalyst metrics but also the pathway’s location on the cost-carbon plane under explicit assumptions, utilizing TEA/LCA to set design goals rather than to defend results after the fact.

Collectively, these sections demonstrate that CO_2_ hydrogenation is not governed by isolated advances in catalysis, data science, or process engineering, but by their integration into a unified framework where mechanistic descriptors guide catalyst design, data-driven tools accelerate discovery, and techno-economic constraints define viable operating windows.

## 8 Conclusions

This review positions CO_2_ hydrogenation as an interconnected reaction network in which product outcomes are governed by selectivity under practical operating and system constraints, rather than by conversion alone. Across RWGS, methanation, methanol synthesis, and C_2_^+^ pathways, selectivity is controlled by recurring mechanistic levers, including CO adsorption and desorption, hydrogen activation and surface coverage, defect and oxygen-vacancy chemistry, oxygenate-intermediate stabilization, and C–C coupling kinetics. These levers are meaningful only when they remain operative under realistic conditions, where heat release, water formation, redox cycling, catalyst restructuring, and nonsteady operation can alter the working active sites and shift product distributions.

Established Ni-, Fe-, and Cu-based catalysts illustrate this framework from a complementary perspective. Ni-based systems highlight the competition between CO desorption and deep hydrogenation to CH_4_; Fe-based catalysts demonstrate how oxide–carbide phase evolution governs RWGS–FTS coupling and C_2_^+^ formation; and Cu-based and mixed-oxide systems emphasize the importance of metal–oxide interfaces, oxygen vacancies, and formate/methoxy stabilization in methanol synthesis. Beyond these benchmark families, emerging catalysts, including carbides, phosphides, intermetallics, high-entropy materials, and multication oxides, expand the accessible design space by introducing new combinations of electronic structure, redox flexibility, ensemble geometry, and defect chemistry. Their value, however, should be assessed not only by peak activity or single-pass selectivity, but by the durability of selectivity under steam-rich, thermally demanding, and dynamically changing reaction environments.

Operando and time-resolved measurements are therefore central to future catalyst development. These tools reveal the working catalyst state, distinguish active intermediates from spectator species, and track phase evolution, redox cycling, sintering, carburization, oxidation, or vacancy dynamics during reaction. Such information provides the physically meaningful descriptors needed for data-driven catalyst design. AI and machine-learning approaches will be most impactful when they move beyond activity prediction to incorporate descriptors of selectivity, stability, catalyst reconstruction, intermediate coverage, and process-relevant operating windows.

At the process level, selectivity is further shaped by transport and energy-transfer phenomena. Temperature gradients, residence-time distributions, heat removal, local H_2_/CO_2_ ratios, water management, and mass-transfer limitations can redirect reaction pathways, particularly in highly exothermic methanation and multistep C_2_^+^ synthesis. Catalyst–reactor–separation codesign is therefore essential for deployment. Techno-economic and life-cycle assessments provide common boundary conditions that link chemistry to scale, consistently showing that hydrogen supply, electricity carbon intensity, CO_2_ source, separation duty, recycle ratio, and heat integration determine whether a selective catalyst can deliver a meaningful environmental benefit. Future progress in CO_2_ hydrogenation will require these constraints to be used not only to evaluate final processes, but also to define catalyst design targets from the outset. This integrated approach can translate mechanistic insight into deployable, environmentally meaningful CO_2_ conversion technologies.
